# Strigolactones and antifungals induce internalization of major facilitator superfamily transporters

**DOI:** 10.1016/j.jbc.2026.113631

**Published:** 2026-10-09

**Authors:** George Ly, James M. Bradley, Freddie R. Montague, Michael Bunsick, Christopher S.P. McErlean, Peter McCourt, Dario Bonetta, Shelley Lumba

**Affiliations:** 1 Department of Cell & Systems Biology, University of Toronto, Toronto, Ontario, Canada; 2 School of Chemistry, The University of Sydney, New South Wales, Australia; 3 Centre for the Analysis of Genome Evolution and Function, University of Toronto, Toronto, Ontario, Canada; 4 Ontario Tech University, Oshawa, Ontario, Canada

**Keywords:** transporter, yeast genetics, molecular dynamics, plant hormone, drug transport

## Abstract

The major facilitator superfamily (MFS) is a diverse class of selective transporters that move a range of substrates across membranes. Despite their central role in cellular homeostasis, chemically perturbing MFS transporters is challenging. Here, we elucidate how the plant hormone, strigolactone (SL), internalizes the yeast MFS phosphate transporter, Pho84, and discover that the agricultural fungicide, chlorothalonil, also interacts with the SL allosteric binding site to cause Pho84 internalization. This piggybacking mechanism for chlorothalonil not only has agricultural applications but also explains why yeast MFS deletion lines show decreased sensitivity to various small molecule perturbagens. Deletion of the MFS inositol transporter, *ITR1*, for example, results in resistance to specific antifungal azoles. We demonstrate that the Itr1 protein is internalized by these antifungals. These findings propose a new paradigm by which small molecules regulate MFS trafficking and offer new insights to identify drugs targeting MFS transporters.

Successful cellular growth requires controlled chemical exchange between cells and their external environments. Membrane transporters are critical gatekeepers in regulating this ebb and flow of compounds in and out of the cell. Transporters in the Major Facilitator Superfamily (MFS) are central to moving diverse small molecules and, in some cases, efflux waste byproducts to and from cells. This functional diversity, together with their involvement in disease, makes MFS transporters attractive targets for therapeutic intervention 1, 2, 3. Moreover, transporters often pump drugs out of cells, making them important pharmacogenetic targets 4, 5, 6, 7, 8, 9, 10.

Although the function of MFS transporters makes them attractive drug targets, small molecules that perturb these transporters are underrepresented (2). Despite MFS transporters encompassing over 10,000 proteins across all kingdoms, they only share 12 to 18% sequence conservation, making transferring insights between studies challenging 11, 12, 13, 14. Consequently, MFS transporters are categorized by a canonical MFS protein-fold comprising two transmembrane (TM) helical bundles (TM1-6, TM7-12) connected by a flexible cytoplasmic loop. These helices allow substrates to enter the transporter in its outward occluded conformation, and a subsequent rocking motion results in an inward occluded conformation, expelling the substrate to the inside (Fig. S1, 13, 14). Drugs that target MFS transporters interfere with their rocking motions or are orthosteric inhibitors 2, 3.

Aside from moving substrates, many MFS transporters cycle to and from the plasma membrane (PM) as cells adapt to nutrient fluctuations (15). Although transporter cycling is ubiquitous across kingdoms, we know the most from *Saccharomyces cerevisiae* (yeast) (16). MFS transporters internalize as substrate levels change. In yeast, high levels of external inorganic phosphate (Pi) cause endocytosis of the high-affinity MFS Pi transporter, Pho84 (17). Similar responses are documented for MFS transporters of sugar, monocarboxylates, amino acids, nucleosides, and micronutrients (1). How substrate concentrations initiate internalization is unclear, but ubiquitination and phosphorylation are often necessary to produce endosomes (16). Once formed, early endosomes either cycle back to the PM or become late endosomes targeted to the Trans-Golgi Network or the vacuole.

Pho84 is subject to regulation at different levels: transcriptionally through the *PHO* pathway or post-translationally through protein kinase A (PKA)-mediated endocytosis. Furthermore, there is evidence that Pho84 functions as a transceptor (18) both transporting phosphate and activating PKA signaling in response to external phosphate 19, 20. This process leads to a self-limiting cascade in which Pho84 effectively signals for its own removal from the plasma membrane 21, 22. For example, when phosphate is added to phosphate-starved cells, Pho84-stimulated PKA signaling drives phosphorylation and ubiquitination of residues within a large intracellular loop in Pho84. These modifications are the molecular trigger for internalization and vacuolar targeting, without them, Pho84 stays at the membrane (22). This is supported by genetic and pharmacological evidence which places PKA activity upstream of endocytosis.

Indeed, strains with attenuated subunits of the PKA complex, Takashi's Protein Kinase (*tpk*) alleles, or those treated with the PKA inhibitor, H89, show significantly delayed internalization, retaining elevated phosphate uptake activity well beyond the wild-type window (22) due to persistence of Pho84 at the plasma membrane. PKA inhibition also disrupts post-endocytic sorting *via* the multivesicular body (MVB) pathway, indicating that PKA activity is required not only to initiate internalization but also to mediate Pho84 movement to the vacuole for degradation 21, 23. Among the three catalytic subunits of the PKA complex, Tpk3 is most important. In strains lacking functional Tpk3, internalized Pho84 fails to reach the vacuolar lumen and instead accumulates in non-vacuolar endosomal compartments (22).

Attenuated Tpk1 and Tpk2 delay internalization, though Tpk2 is generally the most active subunit for initial PKA activation by phosphate (22). The specific requirement for Tpk3 downstream suggests the three subunits have functionally distinct roles that extend well beyond their relative catalytic contributions. Finally, although PKA drives the physical removal of Pho84 from the membrane, it is dispensable for transcriptional repression of the *PHO* pathway. H89 treatment delayed Pho84 degradation without affecting downregulation of secreted acid phosphatase; a transcriptional readout of *PHO* pathway output. The two responses are coordinated, but the mechanisms are distinct: phosphate repletion triggers both a fast, PKA-driven post-translational response at the membrane and a slower transcriptional shutdown through the *PHO* pathway.

Teasing apart MFS internalization and endocytosis is difficult because components are redundant, hence genetic phenotypes are often masked (24). Recently, we discovered that a plant-derived small molecule signal for fungi (25), strigolactone (SL), allosterically binds a transmembrane pocket on Pho84, targeting it to the vacuole. Here, we show the SL transmembrane pocket is cryptic, only forming features when SL is present. SL causes conformational changes in the central cytoplasmic loop of Pho84, which likely signals its internalization. Notably, phosphorylation of this central loop is required for SL-dependent Pho84 internalization.

The identification of a transmembrane SL binding domain on Pho84 is intriguing as natural genetic variation in this pocket also underlies resistance to antifungal agents such as polychlorinated phenols (PCPs) (26). Why polymorphisms at the SL binding site affect PCP sensitivity is unclear, but the internalization of Pho84 by SL suggests that other small molecules interact with this cryptic pocket and internalize Pho84. Importantly, by internalizing Pho84, these compounds can piggyback onto transporters to enter cells and affect internal cellular processes. If true, pharmacogenetic variation that alters compound binding on a transporter could alter compound uptake. Here, we demonstrated that CTN (27) caused Pho84 internalization through a similar mechanism as the plant symbiosis signal, SL. In addition, docking experiments predicted that the SL binding pocket in Pho84 accommodates CTN and other small molecules. This is further supported by *pho84* mutants that confer resistance to both CTN and SL. The relationship of CTN sensitivity and Pho84 internalization explains why MFS deletion mutants in yeast confer resistance to specific small molecule drugs (28).

We found our results could be extrapolated to another MFS transporter, *ITR1. ITR1* encodes the primary inositol MFS transporter in yeast. Similarly to Pho84, Itr1 is regulated at both a transcriptional and post-translational level through endocytosis. When yeast is grown in media replete with inositol, transcription of *ITR1* is repressed through the *INO* pathway 29, 30. In tandem, under high exogenous inositol, Itr1 is rapidly internalized from the plasma membrane and degraded through endocytosis (30). Yeast strains defective in the endocytic pathway or activity of vacuolar proteases were shown to be defective in Itr1 degradation (30). Interestingly, it has been shown this internalization can be perturbed *via* addition of exogenous H_2_O_2_ (31).

We found that deletion of *ITR1* increases resistance to the antifungals ketoconazole and tebuconazole but not clotrimazole (28). These three antifungals are azoles, one of the four classes of antifungal drugs used to treat human fungal infections (32). Ketoconazole and clotrimazole are broad-spectrum antifungals, typically used for the treatment of skin infections (33), while tebuconazole is generally used in an agricultural context (34). Both ketoconazole and tebuconazole inhibit growth by inhibiting ergosterol biosynthesis, although they act at different points in the pathway 35, 36, 37. We demonstrated that addition of tebuconazole and ketoconazole internalized Itr1, but clotrimazole did not. Our results reveal a pipeline to systematically identify small molecules that inhibit MFS transporters through internalization, suggesting a new approach to discovering drugs that target MFS transporter function.

## Results

### SL binds to a cryptic pocket

An SL binding pocket on Pho84 was initially mapped on a Pho84 crystal structure from the fungus, *Serendipita indica* (SiPho84) (Fig. S2*A*, 38, 39). However, an AlphaFold2 (AF2) structure of yeast Pho84 did not show an obvious pocket, while a structure based on AlphaFold3, AF3, possessed a shallow pocket (Figs. S2*A*, (38)). Closer inspection of the SiPho84 crystal (39) revealed a small, positive 2FoFc electron density in the SL binding pocket (Fig. S2*B*). The difference between the Fo (observed electron density) and the Fc (electron density calculated from the structural model) suggested a conformation where the pocket is closed with an unmodelled small molecule inducing a partial opening of the pocket. The appearance of pockets due to crystallization buffer constituents is not uncommon and often uncovers cryptic pockets (40). We hypothesized that the presence of a small molecule will stabilize the pocket region, resulting in a lower B-factor, which is a measure of how much an atom is moving relative to its average position. As predicted, the transmembrane pocket on the SiPho84 structure has a lower B-factor than other amino acids in the transmembrane domains of the protein (Fig. S2*C*).

Cryptic pockets initially appear featureless, but become more pronounced through thermodynamics movements (41). If a favorable conformation appears, a compound can bind to stabilize this state (conformational selection). Alternatively, a conformational change may be “induced” (induced-fit binding) if the compound interacts with the receptor and subsequently induces features to improve further binding (41). To differentiate these models, we performed Molecular Dynamic (MD) simulations on Pho84 to give an atomic-level view of amino acid movements with a ligand. As there is no structure of the entire Pho84 protein, modelling was done using the AF2 Pho84 structure bound with the functionally active synthetic SL isomer, (+)-(*2′R*)-GR24 (+GR24) 42, 43. +GR24 was inserted into the transmembrane domain where the SL-binding pocket was predicted to be (Fig. 1*A*) (38). In the absence of +GR24, the transmembrane pocket was shallow, fluctuating between a closed and partially open state (Fig. 1*B* and Movie S1). The presence of +GR24 stabilized the pocket in Pho84 and increased its overall volume (Fig. 1, *B* and *C* and Movie S1). Similar MD analysis using the non-naturally occurring SL isomer that does not induce a Pi response in yeast, (−)-(*2′S*)-GR24, did not result in a stabilized pocket (Fig. S3*A*).

**Figure 1:**
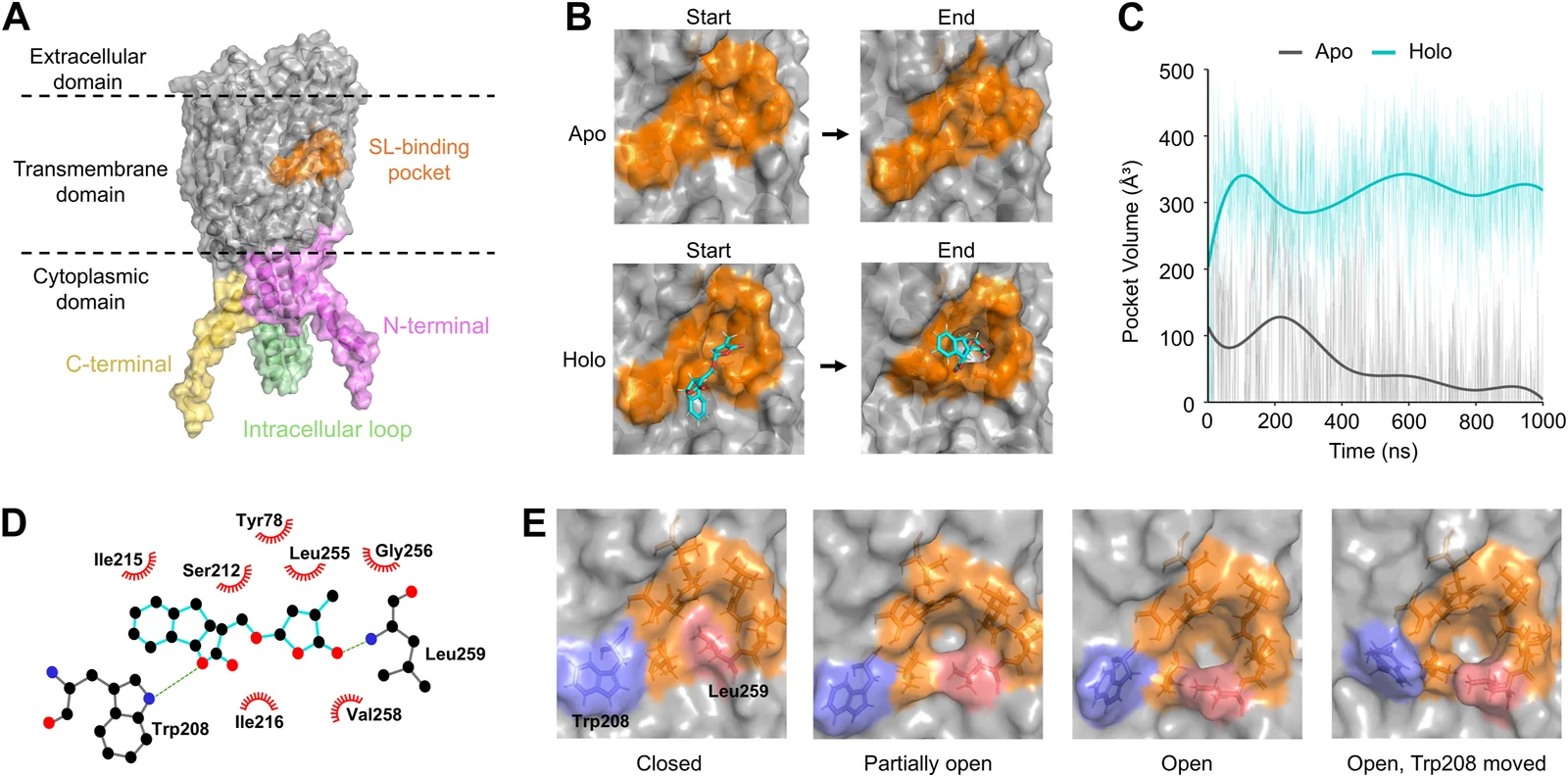
**Analysis of the Pho84 cryptic pocket using Molecular Dynamics (MD) simulations.***A*, diagram depicting domains of Pho84 on the AlphaFold2 predicted structure. The strigolactone (SL) binding pocket is coloured in *orange*. Cytoplasmic domains are coloured: N-terminal domain (*purple*), intracellular loop (*green*), C-terminal domain (*yellow*). (*B*) Start and endpoints of Pho84 MD simulation with no ligand (*top*) or with (+)-(2′R)-GR24 (+GR24) (*bottom*). *C*, volume of +GR24 binding pocket measured across MD simulation. *D*, representation of residues on Pho84 that interact with (+)-(2′R)-GR24 after molecular dynamics simulations. *E*, poses of the SL-binding pocket during molecular dynamics. The closed, partially open, open, and open with Trp208 moved poses are shown. Trp208 is coloured in *purple* while Leu259 is coloured in *pink*.

The Pho84 cryptic pocket consists of Leu74, Tyr78, Trp208, Ser212, Ile216, Leu255, Gly256, Thr257, Val258, and Leu259 (Fig. 1*D*). In the absence of +GR24, Leu259 is pointed toward the pocket region, having a weak hydrophobic bond with Leu74 (Fig. 1*E* and Movie S1). This bond occasionally breaks and Leu74 points outwards, resulting in a slight opening of the pocket (Fig. 1*E* and Movie S1). The D-ring of +GR24 H-bonds with Leu259, resulting in Leu259 being stabilized in an outward-pointing conformation (Fig. 1*D* and Movie S1). This conformational change results in a larger, stabilized pocket. Once the D-ring enters the cryptic pocket, the A-ring of +GR24 is stabilized by a pi-interaction with Trp208 (Fig. 1*D, E*, and Movie S1).

MD analysis of a Pho84 variant (A262P), which reduces yeast SL sensitivity (38), resulted in a shallow binding pocket even in the presence of +GR24 (Fig. S3*B*). Until now, it was difficult to understand how the Ala to Pro substitution resulted in SL insensitivity as this amino acid is at some distance from the SL binding site. A conformational selection model explained the transmembrane pocket transitions between a closed and partially open (∼200 Å^3^) conformation in the apo state (Fig. 1*C* and Movie S1). However, +GR24 not only stabilized but also enlarged the pocket to form a fully open state (∼400 Å^3^) (Fig. 1*C* and Movie S1), supporting an induced fit model. Thus, it appeared that SL binding to Pho84 is best represented by a mixed select and induced model, a scenario that is seen for other proteins containing cryptic binding pockets (41).

### SL induces conformational changes in the cytoplasmic domain of Pho84

SL induces Pho84 internalization under low Pi conditions, which typically cause Pho84 to accumulate in the plasma membrane (38). To identify additional domains in Pho84 influenced by SL binding, we compared the Root Means Square Fluctuation (RMSF), which measures overall protein flexibility, between bound and unbound Pho84 structures. Four regions in Pho84 showed significant movement in the presence of +GR24: the N-terminal, the intracellular loop, a small extracellular region, and the C-terminal domain (Fig. 2*A* and Movie S2).

**Figure 2:**
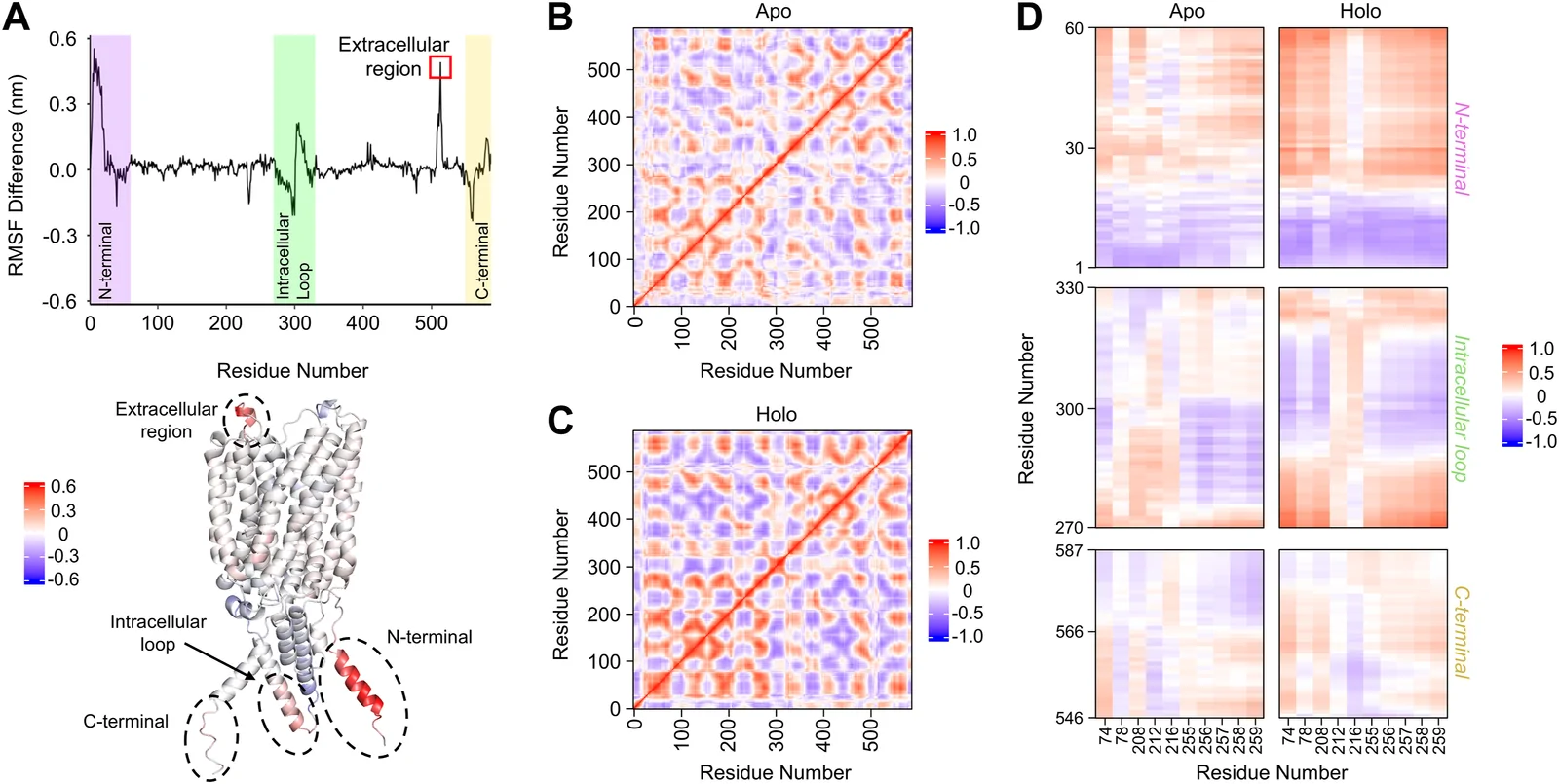
**Analysis of protein-wide dynamics in molecular dynamics simulations of Pho84.***A*, dynamic movement of the domains of Pho84. (*Top*) Difference in root means square fluctuation (RMSF) between +GR24-bound and unbound simulations. Cytoplasmic domains are coloured: N-terminal (*purple*), Inner Loop (*green*), C-terminal (*yellow*). (*Bottom*) Structure of Pho84 coloured based on differences in RMSF. *Red* denotes higher difference and *blue* denotes lower difference between +GR24-bound and unbound simulations. Regions of high difference in RMSF are denoted by dashed circle. *B*, Covariance matrix of residues in Pho84 during molecular dynamics simulations. The colour scale depicts the correlation between two residues at those indices. *Red* indicates higher correlation, white indicates no correlation, and *blue* indicates anti-correlation. *C*, covariance matrix of residues in Pho84 bound with +GR24 during molecular dynamics simulations. The colour scale depicts the correlation between two residues at those indices. *Red* indicates higher correlation, *white* indicates no correlation, and *blue* indicates anti-correlation. *D*, covariance scores between SL-binding pocket residues and cytoplasmic domain residues. Covariance scores between residues within the N-terminal (*top*), intracellular loop (*middle*), C-terminal (*bottom*) domains and residues within the SL-binding pocket domain are shown. Covariance scores from MD simulations generated in the absence of +GR24 (*left*) and in the presence of +GR24 (*right*) are shown. The colour scale depicts the correlation between two residues at those indices. *Red* indicates higher correlation, *white* indicates no correlation, and *blue* indicates anti-correlation.

To further examine the dynamics of the cytoplasmic domain, we performed residue covariance analysis on the trajectories generated from MD simulations of Pho84 to find correlated motions between residues (Fig. 2, *B* and *C*). In the presence of +GR24, there were generally higher correlations between residues on a protein-wide scale (Fig. 2, *B* and *C*). This increased correlation between residues was particularly pronounced in the first 300 amino acids of Pho84 (Fig. 2, *B* and *C*). Since the first 300 amino acids of Pho84 includes the N-terminal, portions of the intracellular loop, and the SL-binding pocket, we examined the covariance between residues in the pocket and residues in all three cytoplasmic domains (Fig. 2*D*). We found that in the presence of +GR24, there were stronger correlated movements between residues in the SL-binding pocket and residues in the N-terminal and intracellular loop compared to residues in the C-terminal domain (Fig. 2*D*).

**Figure 3:**
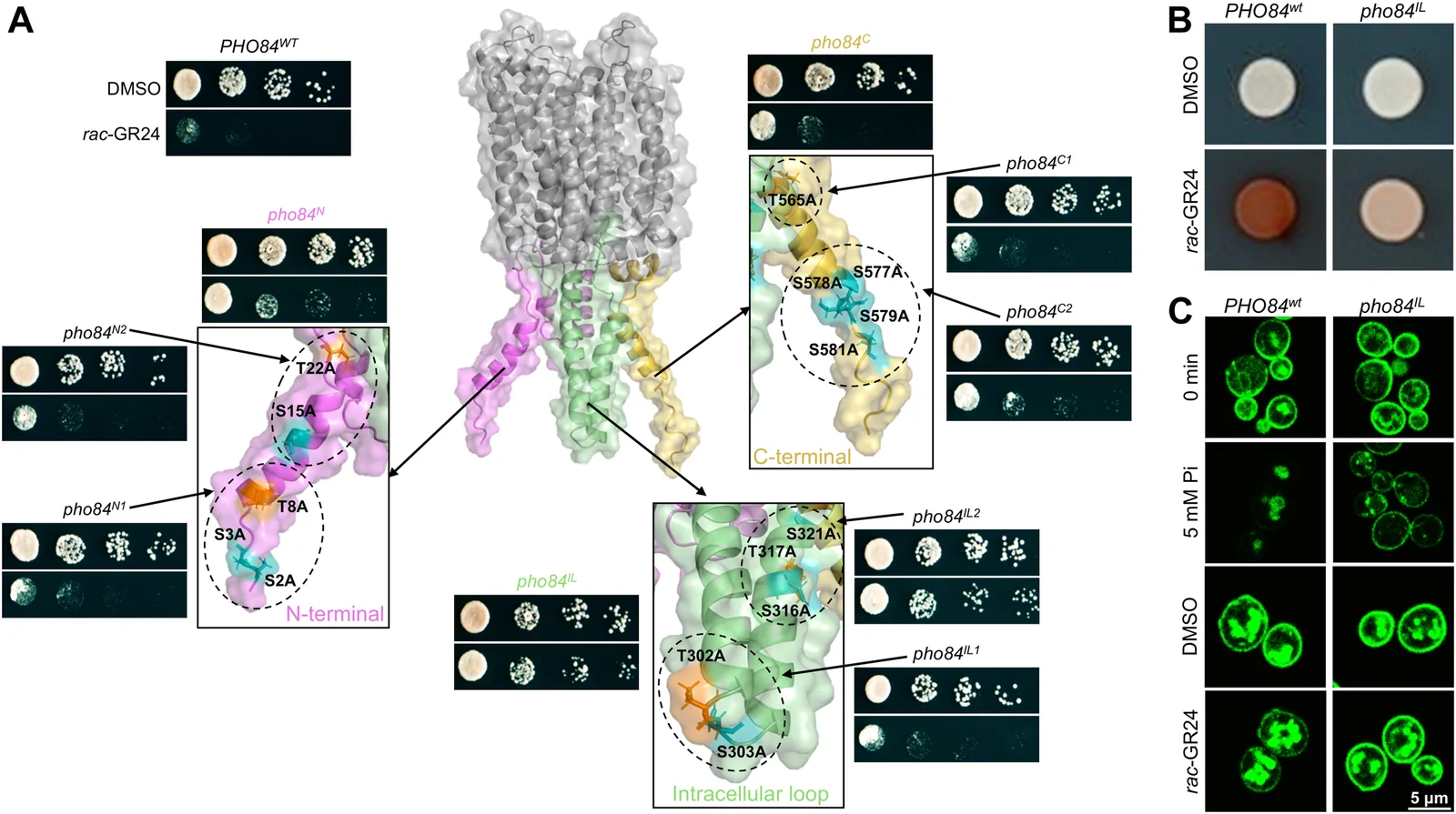
**Phospho-mutational analysis of Pho84 central cytoplasmic loops.***A*, serial growth dilutions of *PHO84* wild type and specific phospho-deficient mutants in the presence of *rac*-GR24. *PHO84*^*WT*^ is a strain (EY917) expressing *PHO84* from a *GAL1* promoter, which has a synthetic lethal phenotype when grown on *rac*-GR24 (38). Each box represents a *PHO84* allele with potential phosphorylation sites mutated to Ala. Growth is shown after 2 days on *rac*-GR24 or DMSO. *B*, acid phosphatase (AP) activity of yeast strains expressing either a *PHO84*^*WT*^ or *PHO84*^*IL*^ allele in the presence of 50 μM rac-GR24 or DMSO. Intensity of red staining reflects AP activity. *C*, Pho84 localization in response to *rac*-GR24. *PHO84*^*WT*^*-GFP* or *PHO84*^*IL*^-*GFP* strains were imaged before and after addition of 5 mM Pi for 60 min, or 50 μM *rac*-GR24 or DMSO with 0.05 mM Pi for 120 min.

To determine large-scale conformational changes resulting from SL binding, we performed principal component analysis of the MD trajectories (Fig. S4). We found that when bound to +GR24, Pho84 underwent distinct conformational changes in the cytoplasmic loop that were not present in the absence of a ligand (Fig. S4, *B*, *C* and *E*). Examining extreme poses along the first two principal components showed that these conformational changes were driven by movement in the N-terminal domain, where the N-terminal moves between being flexible to being bound to the intracellular loop and C-terminal domains (Fig. S4, *C* and *D*). These conformational changes did not occur in the absence of +GR24. Projecting the MD trajectories obtained without +GR24 onto the principal components derived from the +GR24-bound simulations revealed little variance along the first two principal components. (Fig. S4*B*). This indicated that conformational changes which take place in the presence of +GR24 do not occur in the absence of the ligand.

### SL internalizes Pho84

To determine whether conformational changes in the cytoplasmic domain expose potential phosphorylation sites that could contribute to internalization of Pho84, we calculated the Solvent Accessible Surface Area (SASA), which reflects the surface area accessible to solvent for each region. The SASA of multiple potential phosphorylation sites in the internal cytoplasmic loop (Ser303, Ser316, Thr317, and Ser321) and Ser15 in the in the N-terminal domain increased whereas no potential phosphorylation sites in the C-terminal showed a large difference in SASA. (Fig. S5).

These predicted exposures of phosphorylation sites upon SL binding are consistent with previous studies, which suggest the importance of phosphorylated Thr317 and Ser321 to trigger Pho84 internalization in high Pi and deletion analysis (22). To determine whether SL-induced internalization is also dependent on phosphorylation, we performed alanine-scanning mutagenesis of all the Ser/Thr residues within the N-terminus (*pho84*^*N*^: Ser2Ala, Ser3Ala, Thr8Ala, Ser15Ala, Thr22Ala; *pho84*^*N1*^: Ser2Ala, Ser3Ala, Thr8Ala; *pho84*^*N2*^: Ser15Ala, Thr22Ala), the C-terminus (*pho84*^*C*^: Thr565Ala, Ser577Ala, Ser578Ala, Ser579Ala, Ser581Ala; *pho84*^*C1*^: Thr565Ala; *pho84*^*C2*^: Ser577Ala, Ser578Ala, Ser579Ala, Ser581Ala) or the central cytoplasmic loop (*pho84*^*IL*^: Thr302Ala, Ser303Ala, Ser316Ala, Thr317Ala, Ser321Ala; *pho84*^*IL1*^: Thr302Ala, Ser303Ala; *pho84*^*IL2*^: Ser316Ala, Thr317Ala, Ser321Ala). These phosphomutant sets were expressed in a yeast strain (EY917) (44), which is solely dependent on Pho84 for growth, and assayed for growth in the presence and absence of a racemic mixture of ±GR24 (*rac*-GR24) (38). *rac*-GR24 is usually used for functional experiments involving plants and fungi due to cost considerations.

In the absence of *rac*-GR24, growth of all phosphomutant alleles was indistinguishable from the wild type allele (*PHO84*^*WT*^) indicating Pi uptake was not compromised in these genetic backgrounds (Fig. S6). Addition of *rac*-GR24 inhibited growth of *pho84*^*C*^ strains but did not inhibit growth of the *pho84*^*N*^ and *pho84*^*IL*^ strains (Fig. 3*A*). A second phosphomutant strain (*pho84*^*IL2*^) containing Ala substitutions for Ser316, Thr317 and Ser321 residues, was also resistant to *rac*-GR24, narrowing the list of required residues (Fig. 3*A*). When we assayed the activity of the acid phosphatase, Pho5, which is a readout of Pi starvation through SL inhibition of Pho84 38, 45, the *pho84*^*IL*^ strain showed poor *PHO5* induction in the presence of *rac*-GR24, indicating this strain has reduced sensitivity to SL (Fig. 3*B*).

Our phosphomutant analysis suggests that SL does not directly interfere with active orthosteric Pi transport but inhibits Pi uptake by loss of Pho84 from the PM. To test this, we monitored the SL-dependent rate of internalization of both Pho84^WT^ and pho84^IL^. In the presence of *rac*-GR24, pho84^IL^-GFP accumulated to higher levels in the PM compared to wild-type Pho84-GFP (Fig. 3*C*). Unlike the *rac*-GR24 response, high Pi reduced the pho84^IL^-GFP signal from the PM, suggesting that the mechanism of Pho84 internalization due to high Pi *versus* SL differs (Fig. 3*C*).

To determine whether SL-dependent internalization of Pho84 involves the PKA pathway, which was previously determined to be important for Pho84 endocytosis at high Pi levels 21, 22, 23, we monitored Pho84 internalization in the presence of *rac*-GR24 and H89, an inhibitor of the PKA pathway. We did not detect a difference in Pho84 internalization on H89, which suggested that SL-induced internalization of Pho84 does not require the PKA pathway (Fig. S7). In summary, SL binds Pho84 at a transmembrane cryptic pocket and this results in Pho84 internalization from the PM. Internalization requires phosphorylation of the central cytoplasmic loop, which connects the two transmembrane helices that form the canonical Pho84 MFS fold. This evidence is consistent with the prediction from MD analysis that SL binding exposes phosphorylation sites specifically in the central loop.

### Identifying MFS transporter internalizers

SL internalization of Pho84 suggests that small molecules can trigger internalization of MFS transporters. Natural variation studies in *S. cerevisiae* reveal that resistance to a large collection of small molecule perturbagens genetically maps to *PHO84* (26). Intriguingly, this genetic polymorphism is L259P, a key amino acid in the SL binding pocket on Pho84. To test whether drugs can piggyback on transporters to enter cells, we exposed yeast expressing a Pho84-GFP translational fusion to the fungicide, CTN, previously linked to L259P, and found a concentration-dependent loss of GFP signal from the PM with increasing CTN concentrations (Fig. 4*A*). Substitution of proline at L259 in ScPho84 decreased the ability of CTN and SL to internalize Pho84-GFP (Fig. 4*A*). *pho84-L259P* also had decreased sensitivity to CTN (Figs. S8*A*, (26)). Moreover, internalization of the phosphomutant, pho84^IL^-GFP, decreased in response to CTN (Fig. 4*A*). This suggested that internalization of Pho84 by CTN or SL depends on a shared phosphorylation mechanism.

**Figure 4:**
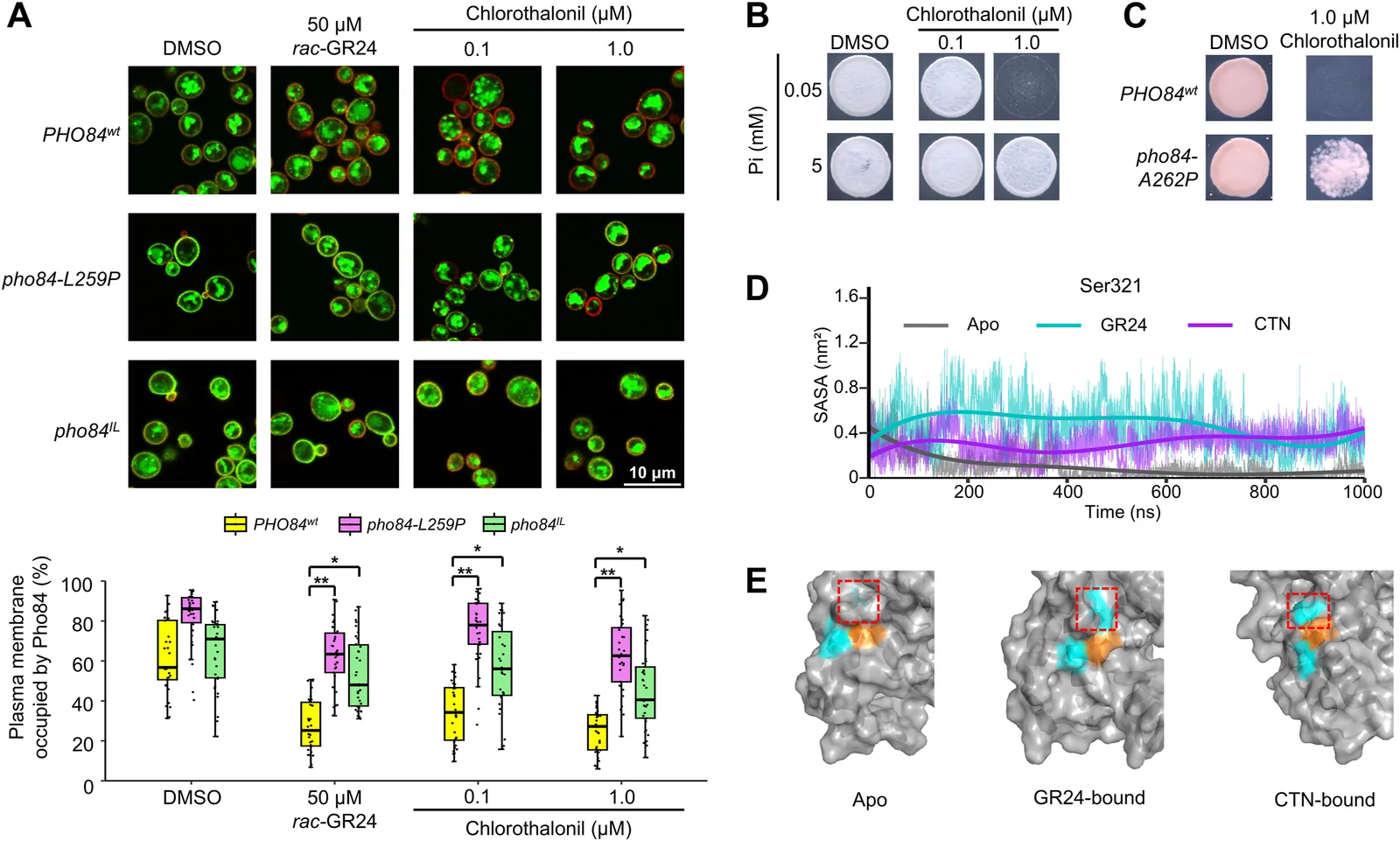
**Effects of chlorothalonil on Pho84 function.***A*, Pho84-GFP and 2xPH-ymScarlet co-localization in wild type, *pho84-L259P*, and *pho84*^*IL*^ yeast upon 4 h of Pi starvation followed by DMSO, 50 μM rac-GR24, 0.1 μM CTN, or 1 μM CTN treatment for 2 h. Each individual datapoint represents a single yeast cell. Two-tailed *t* test was used to measure statistical significance between DMSO and treatment of ligand: ∗ *p* value < 0.05 ∗∗*p* value < 0.005. *B*, wild-type yeast grown on 0.05 mM and 5 mM Pi in the presence of DMSO or CTN. *C*, wild-type or *pho84-A262P* yeast grown on 0.05 mM Pi in the presence of DMSO or CTN. *D*, Solvent-accessible surface area (SASA) of Ser321 on the intracellular loop region in apo, +GR24-, and CTN-bound simulations. *E*, view of the central cytoplasmic loop of Pho84 at the endpoints of apo, +GR24, and CTN-bound simulations. Serine residues are coloured in *blue*; threonine residues are coloured in *orange*. Ser321 is highlighted in a *red-dashed box*.

CTN inhibits oxidative phosphorylation, an internal cellular function (27); thus, yeast sensitivity to this fungicide is a proxy measure of uptake. If yeast growth on CTN is dependent on Pho84 levels, then conditions that deplete this transporter (Fig. 4*B*) should decrease sensitivity to the fungicide. Indeed, yeast grown on 5 mM Pi were less sensitive to CTN inhibition than yeast grown on 0.05 mM Pi (Fig. 4*B*). Additionally *pho84-A262P*, an SL-resistant mutant (38) that we predicted to have a shallow pocket even in the presence of SL (Fig. S3*C*), was less sensitive to CTN (Fig. 4*C*).

MD simulations showed that CTN induced changes in the SL pocket through interactions with the same amino acids that also guide SL binding, including Ser212, Leu255, Leu259, and Ile216 (Fig. S8, *B* and *C*). CTN, like SL, also appeared to move tryptophan at position 208 to anchor the molecule into the pocket (Fig. S8, *B* and *C*). Like SL, CTN induced changes in phosphorylation sites, notably causing increased exposure of Ser321 on the central cytoplasmic loop (Fig. S4, *D* and *E*). Three other Pho84-dependent perturbagens identified by pharmacogenetics (26) also docked into the open pocket pose binding site (Fig. S8*D*). It appeared that the cryptic binding pocket on Pho84 identified through our SL studies can attain conformations that allow binding of numerous small molecules. Binding of these molecules likely causes transporter internalization, which explains the chemical promiscuity of this gene as a chemical hotspot in natural variation studies (26).

Since reducing Pho84 levels decreases CTN response in growth assays, insensitivity of other loss-of-function mutations in MFS transporters to specific small molecule perturbagens could be due to the ability of these small molecules to internalize specific MFS transporters. A deletion of *ITR1 (itr1Δ*), which encodes an MFS transporter involved in inositol uptake, for example, increased insensitivity to azole compounds, tebuconazole and ketoconazole, but not to clotrimazole (Fig. 5*A*) (28). Application of tebuconazole and ketoconazole to a yeast strain containing a translational *ITR1-GFP* fusion, resulted in the internalization of Itr1 but clotrimazole had little effect (Fig. 5, *B*–*D*).

**Figure 5:**
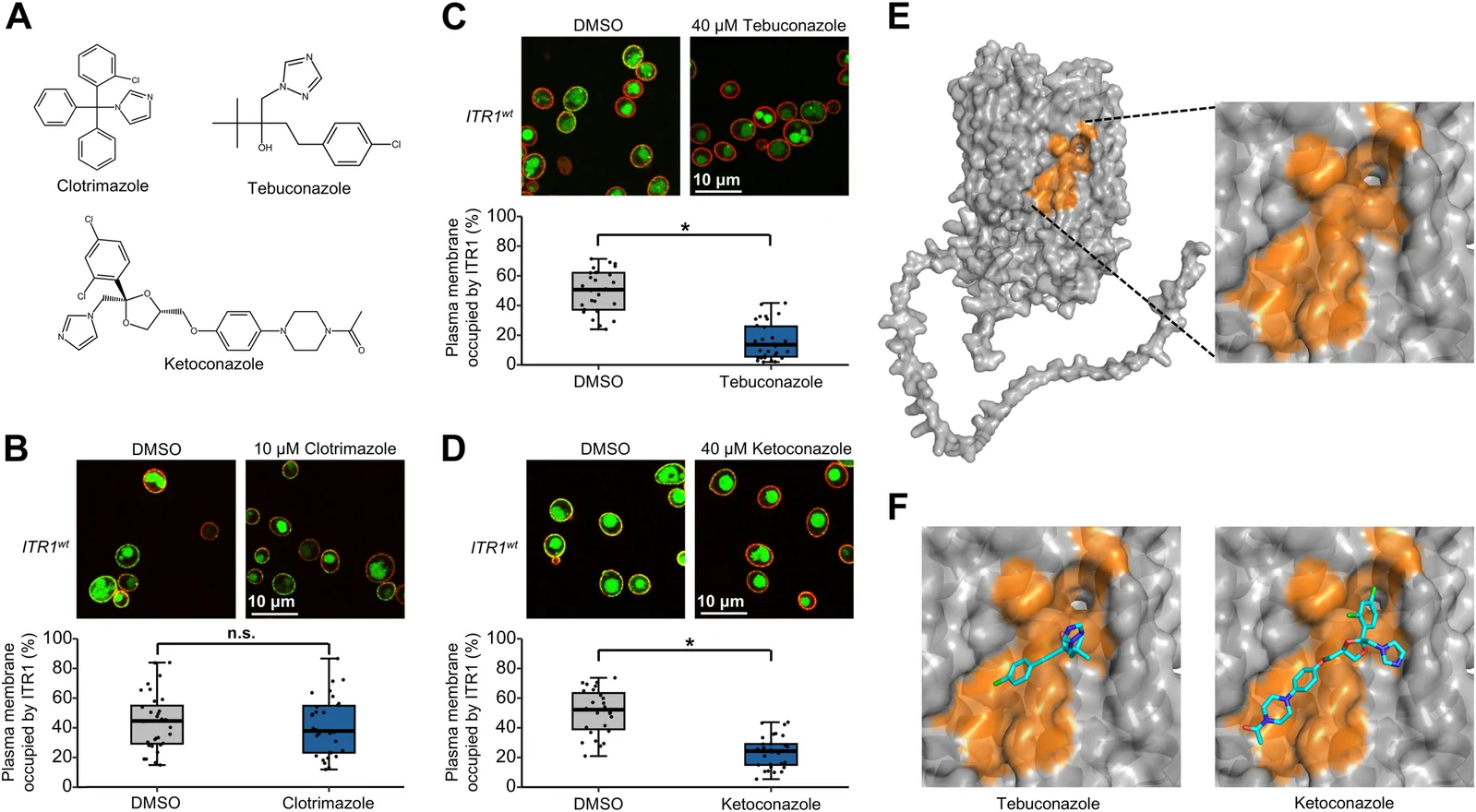
**Effects of specific azoles on localization of the MFS inositol transporter, Itr1.***A*, structures of clotrimazole, tebuconazole and ketoconazole. *B, C, and D*, Itr1-GFP and PM marker, 2xPH-ymScarlet, co-localization following 3 h of inositol starvation and subsequent treatment with (*B*) 10 μM clotrimazole, (*C*) 40 μM tebuconazole, and (*D*) 10 μM ketoconazole. Each individual datapoint represents a single yeast cell. Two-tailed *t* test was used to measure statistical significance between DMSO and treatment of ligand: ∗ *p* value < 0.05. n.s. not significant. *E*, AlphaFold2 structure of Itr1. Predicted binding region of azoles is coloured in orange. *F*, predicted docking poses of tebuconazole and ketoconazole in Itr1.

An AF2-predicted structure of Itr1 contained a transmembrane pocket analogous to the one in Pho84 (Fig. 5*E*). Docking simulations also predicted that both tebuconazole and ketoconazole can bind this transmembrane pocket (Fig. 5*F*). Unlike ketoconazole and tebuconazole which have multiple functional groups, clotrimazole contains only one, a chlorine. Based on our binding analyses of +GR24 and CTN onto the cryptic pocket of Pho84, functional groups (OH on the D-ring of +GR24 and chlorines of CTN) were important for binding the transmembrane binding pocket. The presence of multiple functional groups on ketoconazole and tebuconazole as well as their associations with the Itr1 pocket could explain the ability of ketoconazole and tebuconazole to internalize Itr1.

## Discussion

In this study, we elucidated a previously uncharacterized mechanism by which a plant symbiotic signal, SL, hijacks a Pi transporter, Pho84, in fungi by trafficking this MFS transporter from the PM. The ability of SL to perturb a fungal process is perhaps not surprising as these two kingdoms have interacted since plants became terrestrial (46). The mechanism of SL inhibition, however, is unexpected and offers a promising strategy for the discovery of new drugs that perturb MFS transporters, which is paramount given the limited number of antifungals (47).

We found that SL functions as an allosteric modulator, triggering conformational change in the N-terminal and intracellular domains of Pho84 resulting in increased availability of phosphorylation sites. Since allosteric sites are not conserved between family members, they garner much attention in the pharmaceutical industry as drug targets (48). Orthosteric Pi transport inhibitors, for example, act at mM concentrations on all Pi transporters in yeast, whereas SL interferes only with Pho84 function at much lower levels (38). Second, unlike static binding sites, cryptic pockets only emerge through interaction with a ligand or by dynamic conformational changes. SL therefore offers a new avenue in the development of drugs to dampen MFS transporter activity. Importantly, *PHO84* is homologous to human xenobiotic transporters, which also show drug pharmacogenetic resistance. Possibly, cryptic binding sites contribute to altered drug sensitivity in these human carrier superfamilies.

### A new MFS drug strategy

There are few examples of allosteric drugging of MFS proteins despite their clear therapeutic importance 37, 38. As we demonstrated here, MD simulations of MFS transporters in their native lipid bilayer environment using a natural plant symbiosis compound, integrated with AI-based structural predictions of allosteric binding pockets, and SNP effects, open new opportunities for precise modulation of MFS transporters to develop antifungals.

In this context, previous pharmacogenetic studies in yeast identified a genetic polymorphism within the SL binding pocket of Pho84, which affects yeast’s response to a collection of over 100 small molecules (26). Cryptic pockets, which are dynamically flexible compared to preformed binding sites, increase ligand polyspecificity (49), which suggests that the cryptic pocket of Pho84 contributes to its ligand promiscuity. Our finding that CTN, a completely unrelated molecule to SL, also internalizes Pho84 demonstrates the flexibility of this binding site. It will be interesting to test the ability of other small molecules to internalize Pho84 through this binding motif. Finally, many of the amino acids important for binding SL are highly conserved across Saccharomycetales and in other fungal species suggesting this polyspecific binding pocket may have translational utility (38).

The ability of SL to internalize Pho84 likely resolves the observation where deletions of a number of transporters in yeast improve resistance to seemingly unrelated small molecules (28). MFS deletion alleles in yeast, for example, are resistant to the following small molecules: tebuconazole and ketoconazole (*itr1Δ*), 3,4-dicloroisocoumarin (*flr1*Δ), fluconazole (*dtr1*Δ), 5-fluorocytosine (*qdr*1Δ), 5-fluorocytosine (*qdr*2Δ), and 5-fluorocytosine (*atr1*Δ) (28). A model where small molecules piggyback into cells *via* transporter trafficking explains these resistances. We showed that small molecules like tebuconazole and ketoconazole internalize the Itr1 transporter, suggesting that other molecules can allosterically internalize MFS transporters. Indeed, structural modeling of Itr1 identifies a potential pocket analogous to the Pho84 SL binding pocket. Consequently, chemical screens against a knockout collection of yeast uptake transporters, looking for improved resistance may enrich for such compounds. Secondary screening using collections of translational fusions of these transporters to GFP (50) would quickly determine which compounds function to internalize transporters. The ability to identify small molecules that internalize MFS transporters may not only identify new inhibitors of MFS transport function but also raises the possibility of repurposing small molecules for drug delivery to fungi (51).

The broad polyspecificity of the SL binding site also suggests that compounds binding the Pho84 binding site could be chemical scaffolds to deliver new perturbagens to fungi. The presence of nucleophilic groups on CTN, for example, allows further chemical modifications to obtain novel derivatives with increased potency (52). Broadly, solute carrier transporters including MFS transporters are present throughout the gastrointestinal tract (53). Allosteric internalizer binding sites on these transporters may represent targeting sites for drug delivery that have yet to be exploited.

### Using SL to dissect fungal Pi transport

SL internalization of Pho84 affords opportunities to address questions of Pi homeostasis in yeast and other fungi. Induction of Pi utilization genes under Pi-starvation conditions is not only important for growth but also for recovery after Pi starvation (54). Constitutive expression of *PHO84*, for example, slows the recovery of yeast cycled through between low and high Pi. Directed evolution experiments during Pi cycling identified mutations in Pho84 including L259P, which remarkably, maps to our defined SL binding pocket and predicted to perturb SL binding (54). This suggests that Pho84 positively regulates a yeast-derived SL mimic (Y-SL) that functions in Pi homeostasis. In this model, *PHO84* overexpression results in constitutive Y-SL production internalizing Pho84. Mutations reducing Y-SL binding affinity dampen Pho84 internalization, stabilizing Pi uptake.

The L259P substitution that decreases Pho84 trafficking is prevalent in fungal species suggesting that reduced Pho84 trafficking is a preferred state compared to lab-friendly yeast strains which usually have access to abundant Pi. The existence of Y-SL harkens to the “transceptor” model where Pho84 senses intracellular signals *via* a secondary ligand, rather than Pi itself, to coordinate trafficking and prevent excess accumulation under nutrient-replete conditions (55). Since Pho84 internalization is caused by ligand recognition through a cryptic binding site, trafficking can be finely tuned to various exogenous and endogenous small molecules dependent on both the species and the environment in which they are growing.

Finally, CTN, which we show also internalized Pho84, is a nonsystemic, broad-spectrum crop fungicide (56). In field experiments, CTN interferes with Pi uptake by AM fungi as well as with Pi uptake by host plants (57). Our results explain this finding and predict that in high Pi environments, which are common for a farmer’s field, fungi will be more resistant to CTN due to less Pho84 accumulation in the PM.

## Experimental procedures

### *S. cerevisiae* strains, growth conditions, and media

*S. cerevisiae* (yeast) BY4742 MATalpha strain integrated with a *PHO84*^*WT*^*-GFP* at the native *PHO84* locus was previously described in Bradley *et al.* (2024) (38). A plasmid-borne copy of *PHO84p-PHO84* (*pAG415-PHO84p-PHO84*) with different mutant alleles was introduced into either the EY917 strain (44) or the BY4742 *pho84Δ* strain (38) and selected for using leucine dropout media. The EY917 strain lacks all genomic copies of phosphate transporters, including *PHO84* and harbors a plasmid-born copy of *PHO84* under the control of the *GAL1* promoter, which permits growth on tryptophan dropout media supplemented with galactose. Following the introduction of *pAG415-PHO84p-PHO84*, growth was permitted on media containing glucose as the sole carbon source. Yeast strains were grown in Yeast Nitrogen Base (YNB) without amino acids and without phosphate (Formedium, UK, CYN0804). Amino acids were supplied according to Gietz & Woods (2006) (58). Phosphate was supplied as KH_2_PO_4_ to a concentration of 0.05 mM (low), 0.5 mM (intermediate) or 5 mM (high) phosphate, and K levels were normalized to 10 mM using KCl. *rac*-GR24 was provided by Christopher S. P. McErlean (University of Sydney).

### Construction of phosphomutant Pho84 alleles

Putative serine (S) and threonine (T) phosphorylation sites were identified within the cytoplasmic regions of Pho84 by inspecting the predicted AlphaFold2 model retrieved from the AlphaFold database (59). Cytoplasmic regions were separated into the N-terminal tail (residues 1–50), central cytoplasmic loop (residues 279–346), or the C-terminal tail (residues 552–587). For each region, between one and four putative phosphorylation sites were targeted for mutation to alanine according to their proximity. Specifically, target sites were grouped into clusters according to whether they could be simultaneously mutated using a single pair of mutagenic primers (Tables S1 and S2). A *pAG413m-PHO84p-PHO84* plasmid (38) was used as a template for generating PCR products with mutagenic primers using standard Phusion PCR protocol. Each mutagenic primer was combined with a second primer that bound within the plasmid backbone (Tables S1 and S2). This generated two PCR fragments for each *PHO84* allele, with both fragments having the altered bases incorporated within the *PHO84* primer binding site and each fragment sharing ≥ 30 bp sequence homology. Each fragment also had > 100 bp of homology to the plasmid backbone, either upstream (fragment 1) or downstream (fragment 2). The two PCR fragments plus a pAG415 plasmid backbone (60) linearized with SacI and XhoI, were transformed into yeast and a reconstituted *pAG415-PHO84p-PHO84* plasmid was generated by homologous recombination with desired mutations. Yeast colonies were selected on YNB –leucine agar plates, pooled together and inoculated into 4 ml YNB –leucine media and grown overnight at 30 °C. Plasmid DNA was extracted form 1 ml overnight yeast suspension by first digesting cell walls for 2 h with zymolyase according to manufacturer’s instructions, followed by standard miniprep (NEB Monarch Plasmid Miniprep kit, T1010L). Yeast plasmids were recovered into DH5alpha *E. coli* cells, followed by verification of *PHO84* mutations by Sanger sequencing.

For the *PHO84-IL-Ala* allele, a *GFP*-tagged version of the protein was constructed. First, a previous *PHO84-GFP* strain (38) was used as a template in a Phusion amplification of the C-terminal end of *PHO84* plus *GFP*, using primers ‘PHO84_C’ and ‘pAG_Down_Homology’ (Table S2). Then, the *pAG415-PHO84p-PHO84* plasmid was digested with SacI and NdeI. The PCR product and digested plasmid was co-transformed into BY4742 and the recombined plasmid was selected on YNB –leu agar plates and a colony was selected by screening for Gfp signal under Pi starvation. The plasmid was confirmed by colony PCR and moved into *E. coli*. The *PHO84-IL-Ala-GFP* construct was integrated into the native *PHO84* locus as described in Bradley *et al.* (2024) (38).

### Construction of pho84-L259P

*PHO84-L259P* was engineered using CRISPR-Cas9 gene editing 61, 62. A 20-bp target sequence was chosen which encompassed the desired mutation site and was immediately upstream of a PAM sequence (NGG). Two primers were annealed (making the 20 bp target sequence) and were ligated into the Cas9-containing plasmid, pCAS-Tyr-[gRNA: BsaI GFP dropout] (BsmBI-flanked URA3, AmpR) (63). A 100 bp repair fragment was generated by template-free PCR. The central 20 bp of this fragment was designed to replace the target sequence, while the 40 bp regions flanking either side provide homology to sequences immediately upstream and downstream of the cut site, enabling homologous recombination into the genome. This fragment contained the desired SNP (L259P) as well as two silent mutations. These extra silent mutations were included to prevent the Cas9 from cutting the repair fragment. The guideRNA was then amplified off the Cas9-containing plasmid and was transformed along with BsaI-digested pBBK09, and the repair fragment into a *PHO84-GFP* plasma membrane marker strain (Table S3). The yeast was plated on uracil dropout media to select transformants. The nucleotide was sequenced for confirmation.

### Growth and acid phosphatase assays using *PHO84* phosphomutant alleles

Plasmid borne copies of the *PHO84* phosphomutant alleles were transformed into either the *BY4742 pho84*Δ strain or the *EY917* strain to conduct acid phosphatase or growth assays, respectively. Both assays confirmed the functionality of the *PHO84* phosphomutants as functional Pi transporters. For growth, EY917 strains were grown in 1 ml 5 mM Pi YNB –trp –leu + 2% galactose + 1% raffinose for 3 days to saturation. A 1/5-fold dilution series was spotted onto solid YNB –trp –leu + 2% glucose with different Pi concentrations. Plates were incubated at 30 °C for 36 h prior to imaging. In a separate experiment to determine response to *rac*-GR24, cells were spotted onto YNB –trp –leu + 2% glucose supplemented with 5 mM Pi and 50 μM *rac*-GR24. For acid phosphatase, cells were spotted in the same way onto YNB –leu media supplemented with different concentrations of Pi and, after ∼ 20 h of growth at 30 °C stained for acid phosphatase activity as described in Bradley *et al.* (2024) (38).

### Confocal imaging of yeast strains

*PHO84-GFP* strains were imaged in various yeast strains following the method described in Bradley *et al.* (2024) (38). Briefly, cells were inoculated into 4 ml YNB -leu and grown overnight at 30 °C for ∼ 18 h. Cells were washed in dH_2_O and then resuspended in 0.05 mM Pi YNB-leu medium with 4% glucose for 4 h. Following this, cells were treated with either 5 mM Pi or without Pi addition, but with either DMSO or 50 μM *rac*-GR24. Cells were imaged using a SP5 confocal microscope.

### Transformation of plasma membrane marker into yeast

Plasma membrane marker (2xPH) added to yeast by co-transforming pBBK11 (AatII and SdaI) and pBBK75 (NotI) using the lithium acetate method. After transformation protocol, yeast was recovered in YPD overnight and then plated on YPD + 500 ug/ml G418. Colonies were confirmed by confocal microscopy.

### Quantification of co-localization

Co-localization of Pho84-GFP and Itr1-GFP with a ymScarlet-tagged plasma membrane marker (2xPH) (63) was done using Fiji (64). Images were converted to an 8 bit grayscale, threshold values were then adjusted to isolate pixels of interest while minimizing background fluorescence. From this a composite image was generated, isolating pixels that were overlapping in both the green and red channels. The “analyze particles” function was used to calculate the total pixel areas for both the composite and red channels. Percent of plasma membrane occupied by GFP was generated by calculating a ratio of total pixel area in the composite to total pixel area in the red channel. Graphs of quantified co-localization were generated using the ggplot2 R package 65, 66.

### CTN inhibition assays

Yeast was grown overnight in YNB medium containing 5 mM phosphate. The following day cells were washed with water and resuspended in YNB containing 0.05 mM of phosphate to an OD600 of 1.6. Cultures were then incubated for 4 h then plated on media containing either 0.05 or 5 mM Pi supplemented with DMSO, 0.1 μM CTN, or 1 μM CTN. Pictures were taken after 2 days of growth using a Leica MZ16 microscope.

### Chemical docking

Docking of chemical compounds into the structures of Pho84 and Itr1 was performed using AutoDock Vina (67). A search space of the entire protein was used for docking with a maximum energy range of 3 kcal/mol. An exhaustiveness of eight and random seeds were used for pose generation. The best binding pose was chosen based on lowest binding energy. PyMoL was used to determine no steric clashes occurred (68). The structures of Pho84 and Itr1 were retrieved from the AlphaFold database (59). A bounding box for docking into Pho84 was created based on the interacting amino acids described previously (38). Compounds were docked into a bounding box in a homologous region on Itr1. The top-binding pose for each compound was selected based on the lowest Gibbs free energy, with a cutoff of −1 kcal mol^−1^. PyMOL was used to ensure there were no clashes in the chosen docking poses (68).

### MD simulations

MD simulations of Pho84 in the presence of +GR24, −GR24, and CTN were performed in triplicate cycles of 1 μs using GROMACS (version 2021.4) (69). Each cycle was performed as an independent replicate, assigned a different initial velocity from the same starting coordinates. Starting coordinates for these simulations were obtained by docking as described in the *Chemical docking* section. All systems were prepared using CHARMM-GUI (70). The CHARMM36 force field was used (71). Amino acids were protonated based on a pH of 7.0. The system was constructed by inserting Pho84 into a 1,2-di-O-myristoyl-sn-glycero-3-phosphocholine (DMPC) bilayer, the transmembrane domain was predicted using the PPM web server (72). All protein-bilayer systems were fully solvated and neutralized with a 0.15 M NaCl water solution.

The systems were initially minimized using 5000 steps steepest descent to reach a minimum energy of 1000 kJ mol^−1^ nm^−1^. To obtain canonical ensembles (NVT), minimized systems were heated to 303.15 K over 0.125 ns and then another 0.125 ns for T_m_ equilibration using Velocity Rescale Thermostat. To obtain isothermal-isobaric ensembles (NPT), the system was equilibrated for 1.25 ns at constant pressure using the C-rescale barostat for pressure coupling with semiisotropic position scaling, with a reference pressure of 1.0 bar. Following equilibration, a production simulation was performed on all systems for 1.0 μs. All H-bonds were constrained using the LINCs algorithm, and long-range electrostatic forces were calculated using the Particle-Mesh Ewald (PME) method. A time step of 2 fs was used throughout the production simulation.

To process the trajectories, the protein-ligand systems were recentered in the unit cell. Ligand-binding pocket volume was calculated using MDPocket (73). A voxel size of 2.5 Å was used to calculate pocket volumes. RMSF of the α-carbon backbone throughout the simulation was calculated the rmsf module. SASA was calculated using the sasa module. A probe radius of 0.14 nm was used to calculate SASA. Covariance scores between residues were calculated using the covar module on the α-carbon backbone. Heatmaps were generated using the ComplexHeatmap R package 65, 74. Principal component analysis of α-carbon backbone residues was performed using the anaeig module to output 2d projections of trajectories on principal components and output extreme frames along principal components. Amino acids that interact with the ligand were calculated using LigPlot+ (75). All protein-ligand system visualizations were done using PyMOL (68).

## Data availability

All yeast strains and data created in this study are available upon request. Starting coordinates of all molecular dynamic simulations and AF3 output with penalties are available in Zenodo, https://doi.org/10.5281/zenodo.22800948.

## Supporting information

This article contains supporting information.

## Conflict of interest

The authors declare that they have no conflicts of interest with the contents of this article.
