# Supplementary material for: Strigolactones and antifungals induce internalization of major facilitator superfamily transporters
Source: J Biol Chem. 2026 Oct 9;302(12):113631. doi: 10.1016/j.jbc.2026.113631 (PMC13769216; doi:10.1016/j.jbc.2026.113631)
Supplement: Supplementary file 1 [file mmc2.pdf]

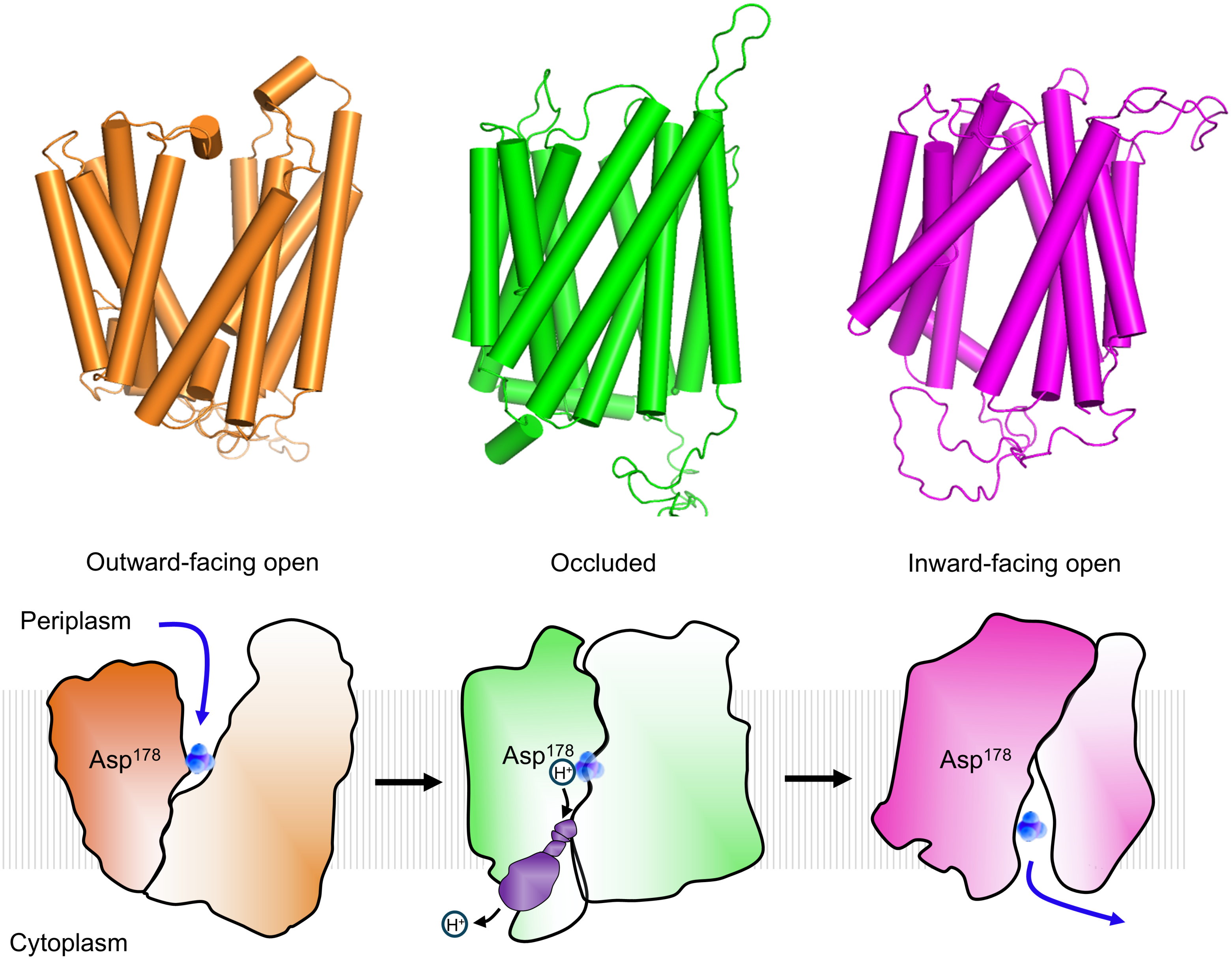

Figure S1. Proposed model of Pho84p phosphate transport. The Pho84 transporter (587 amino acid residues) encompasses 12 transmembrane spanning domains arranged in two bundles of six helices. Based on a classic MFS "rocker-switch" transport mechanism Pho84p can be modelled in three conformations; Outward-facing open (left-brown), Occluded (middle-green), and Inward-facing open (right-pink) based on structural alignments to the *E. coli* fucose symporter (PDB 3O7Q), the *S. indica* high-affinity phosphate transporter, PiPT (PDB 7SP5), and *E. coli* glycerol-3-phosphate transporter (PDB 1PW4), respectively. Pho84 is a proton symporter having residues involved in binding the negatively charged phosphate ion ( ) and a positively charged proton (H+). In a rocker-switch scenario, the protonation of Asp178 closes the substrate binding site to encourage phosphate binding. This opens a negatively charged cytosolic tunnel (purple) in the occluded state where the proton is extruded. The resulting repulsion between phosphate and the now deprotonated binding site allows phosphate to exit to the cytosol between the two domains (Inward-facing open).

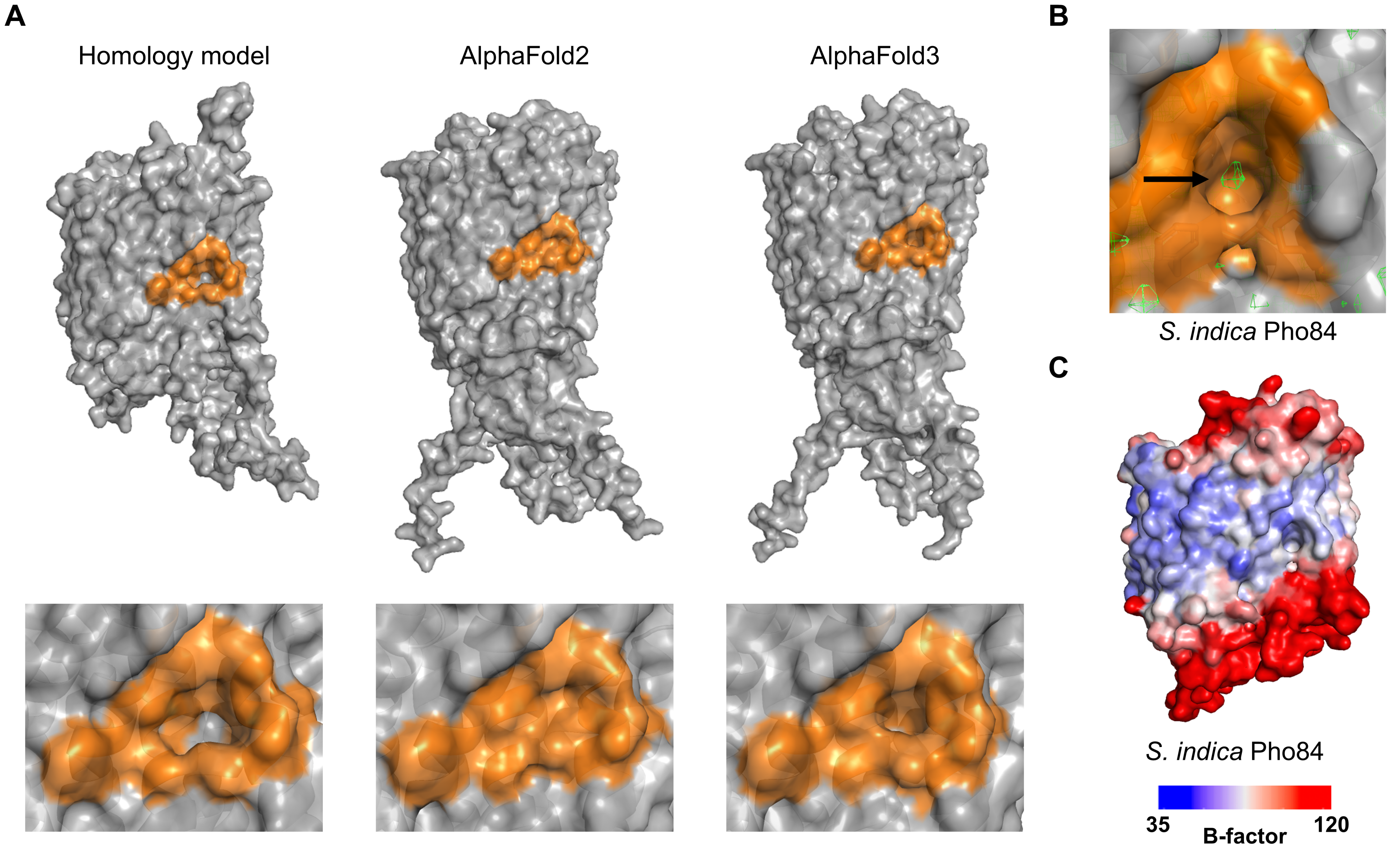

Figure S2. Structural analysis of the Pho84 binding pocket. (**A**) Comparison of different models of Pho84. (Left) Homology model of Pho84, (Middle) AlphaFold2 model of Pho84, (Right) AlphaFold3 model of Pho84. Transmembrane SL-binding pocket is coloured in orange. (Bottom) Close-up view of SL-binding pocket in each model. (**B**) 2FoFc electron density map of the Pho84 SL binding pocket. Arrow points out an unresolved small positive density. (**C**) Protein structure of *S. indica* Pho84 (PDB: 7sp5) coloured in by B-factor. Low B-factors are blue, while high-B-factors are red.

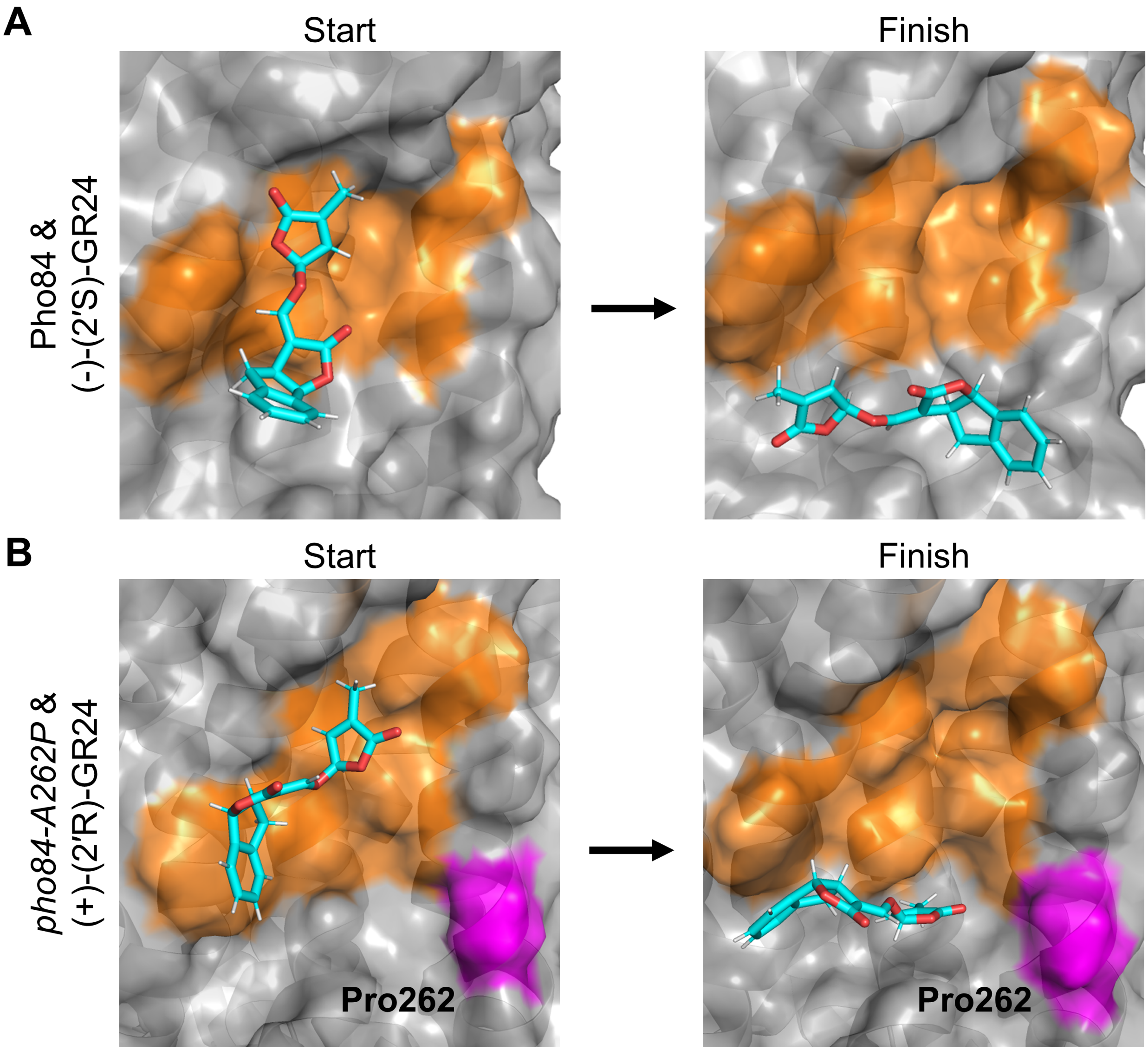

Figure S3. Molecular dynamics analysis of Pho84 with (-)-(2′S)-GR24 and *pho84-A262P.* (**A**) The start and end of a Molecular Dynamics (MD) run involving Pho84 with the non-natural isomeric form (-)-(2′S)-GR24. (**B**) The start and end of a Molecular Dynamics (MD) run involving the mutated Pho84 (Ala262Pro) with a natural isomeric form (+)-(2′R)-GR24. Pro262 is labelled in magenta.

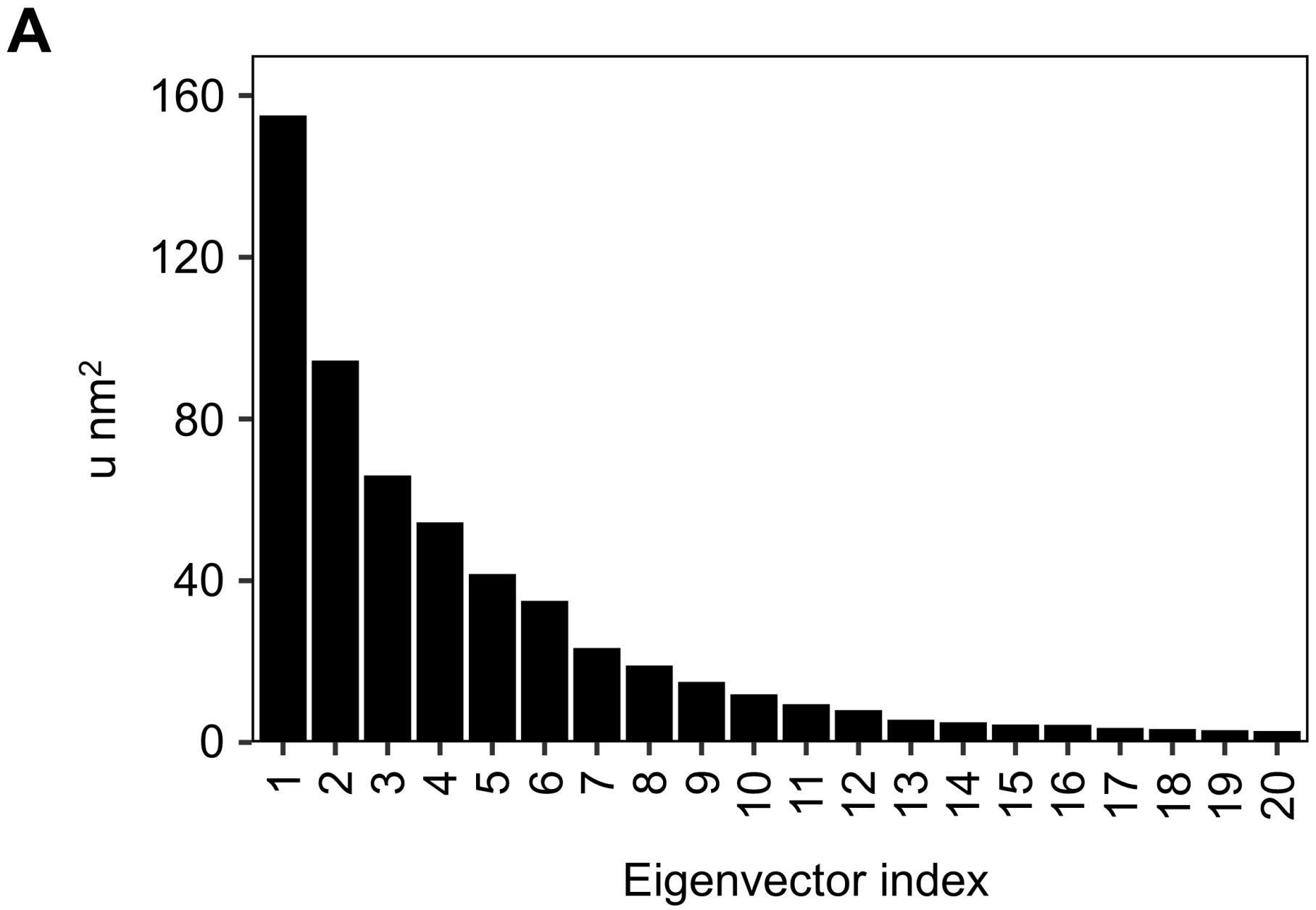

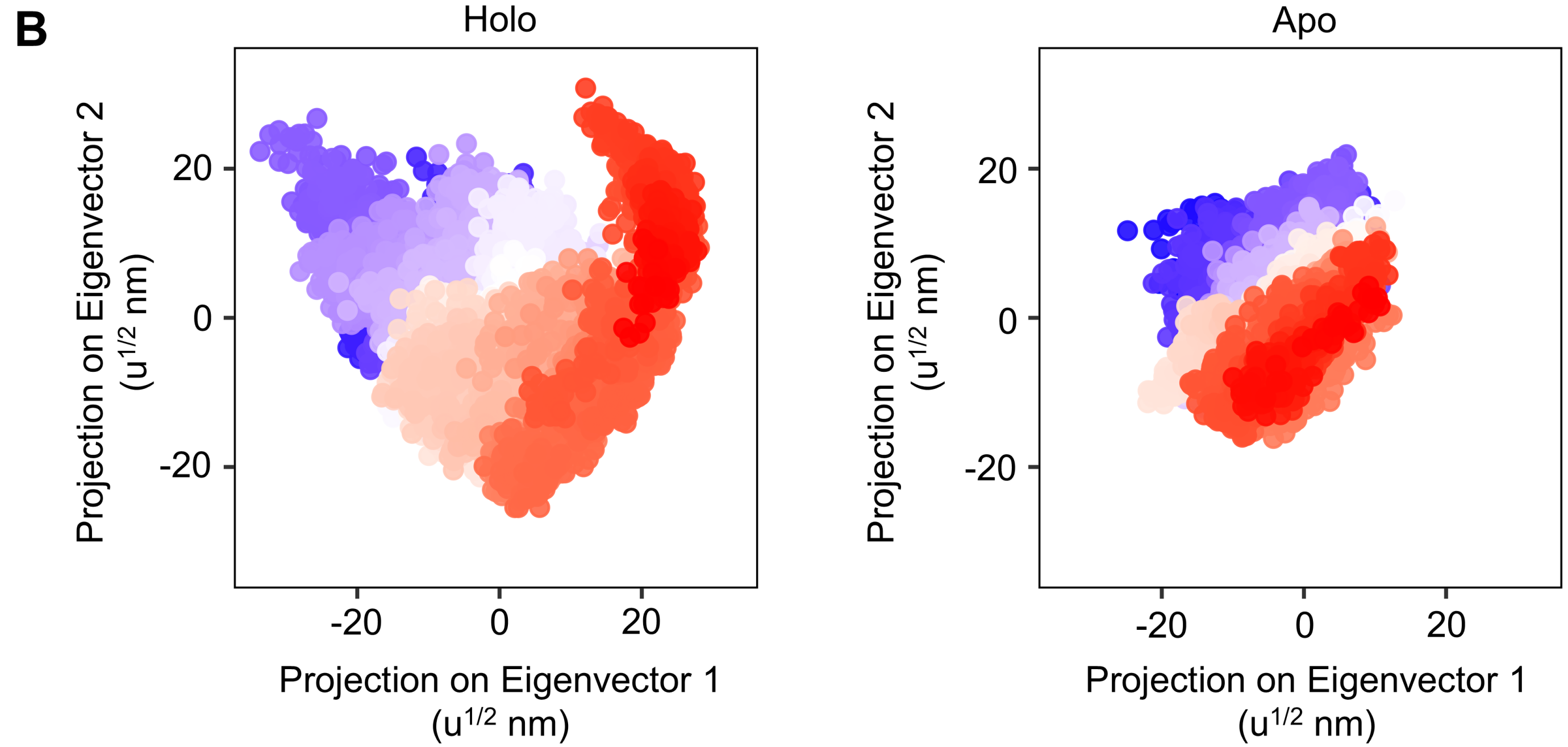

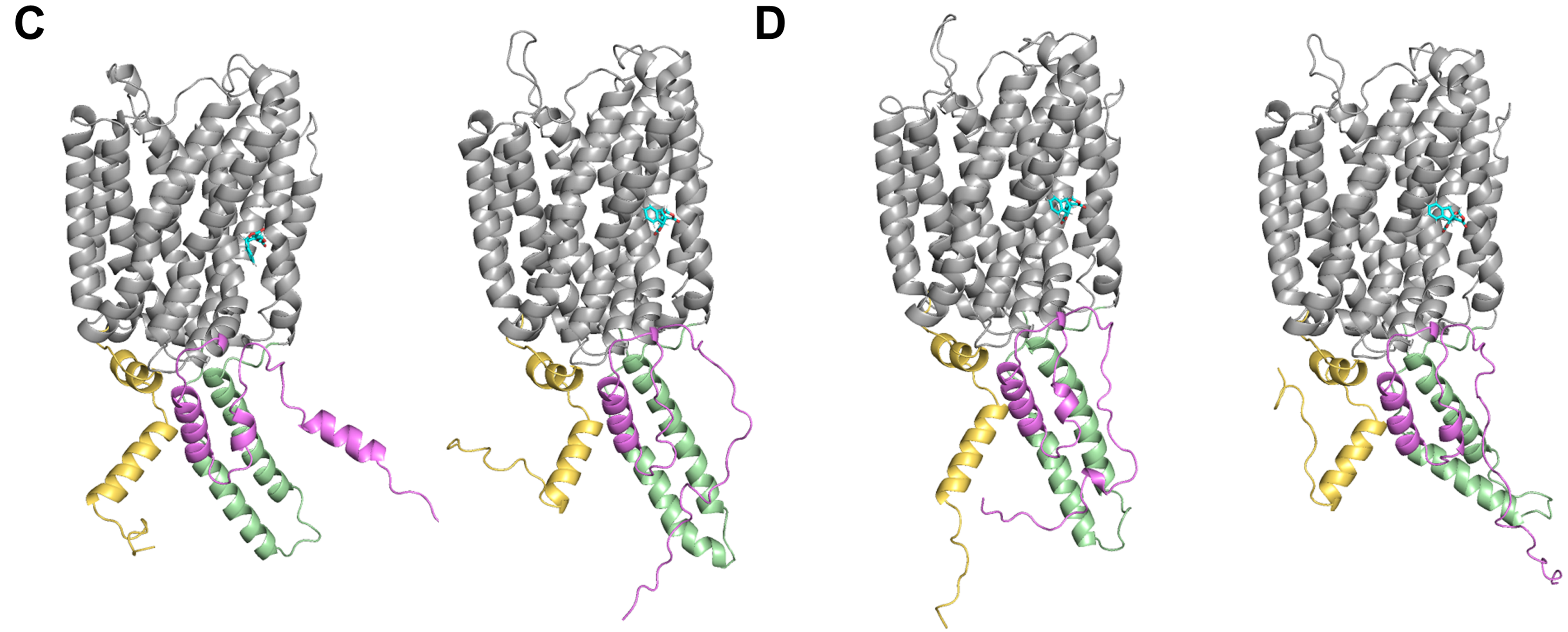

Figure S4. Principal component analysis of Pho84. (**A**) Eigenvalues of the first 20 eigenvectors computed from molecular dynamics simulations of +GR24 bound Pho84. (**B**) Trajectory frames of +GR24 bound Pho84 (Left) or Apo Pho84 (Right) projected onto the first principal components computed from +GR24 bound Pho84 simulations. The colour denotes time in the simulation, with blue denoting earlier timepoints and red denoting later timepoints. (**D**) Extreme structures of +GR24 bound Pho84 along eigenvector 1. Cytoplasmic domains are coloured: N-terminal (purple), Inner Loop (green), C-terminal (Yellow). (**E**) Extreme structures of +GR24 bound Pho84 along eigenvector 2. Cytoplasmic domains are coloured: N-terminal (purple), Inner Loop (green), C-terminal (Yellow).

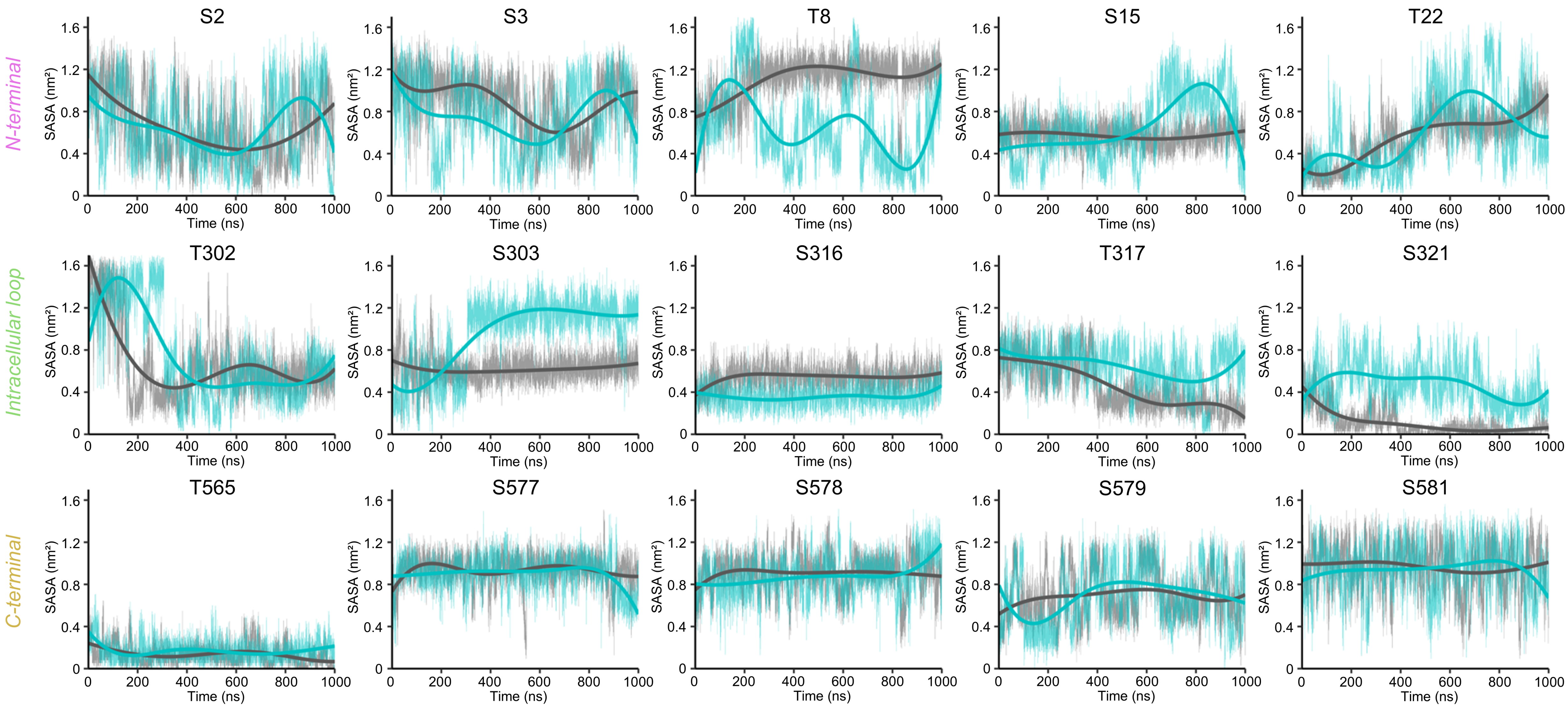

Figure S5. Solvent accessible surface area analysis of the intracellular region of Pho84

Solvent accessible surface area (SASA) of threonine and serine resides located in the intracellular regions of Pho84. Higher values of SASA indicate more surface accessibility in that residue denoting higher availability to interacting proteins and/or solvent. Regions are separated into N-terminal (top), intracellular loop (middle), and C-terminal (bottom). Grey lines represent simulation with no ligand present and blue lines represent simulation with (+)-(2′R)-GR24 present.

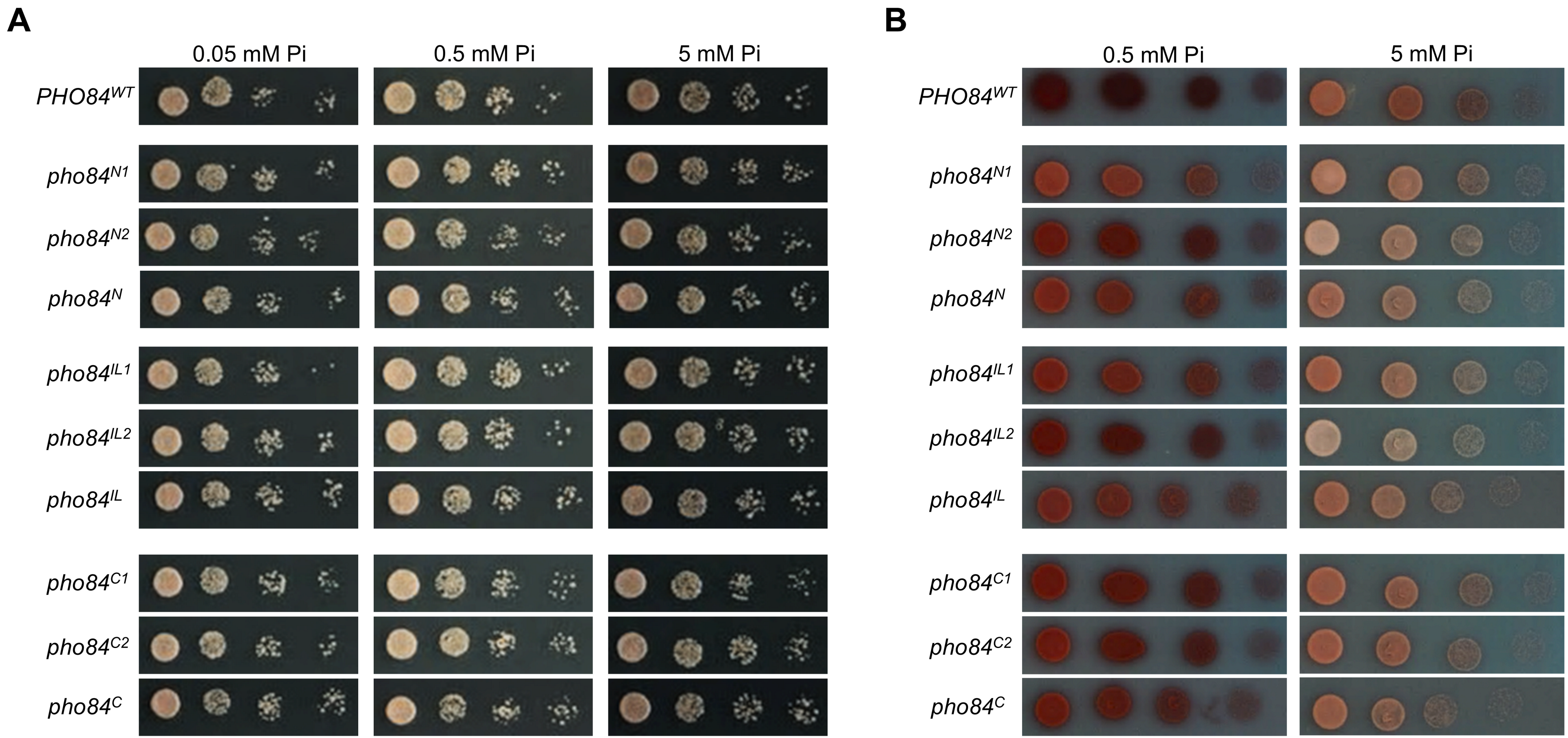

Figure S6. (**A**) EY917 transformed with *pho84* phosphomutant alleles. Transformants were grown in 5 mM Pi SC –trp –leu + galactose for 3 days to saturation. A 1/5 dilution series was spotted onto SC –trp –leu + glucose with different Pi concentrations. Images taken at ~36 hrs. All phosphomutants were expressed under native promoter from a pAG415 vector backbone. (**B**) The same phosphomutant alleles were transformed into BY4742 *pho84*Δ strain and selected on SC –leu. The strains were grown in 5 mM Pi SC -leu for 48 hrs. Then, a 5-fold dilution series was made in dH_2O and 2 µl spotted onto YNB –leu with 0.5 or 5 mM Pi. Cells were grown for ~20 hrs, then stained for AP activity.

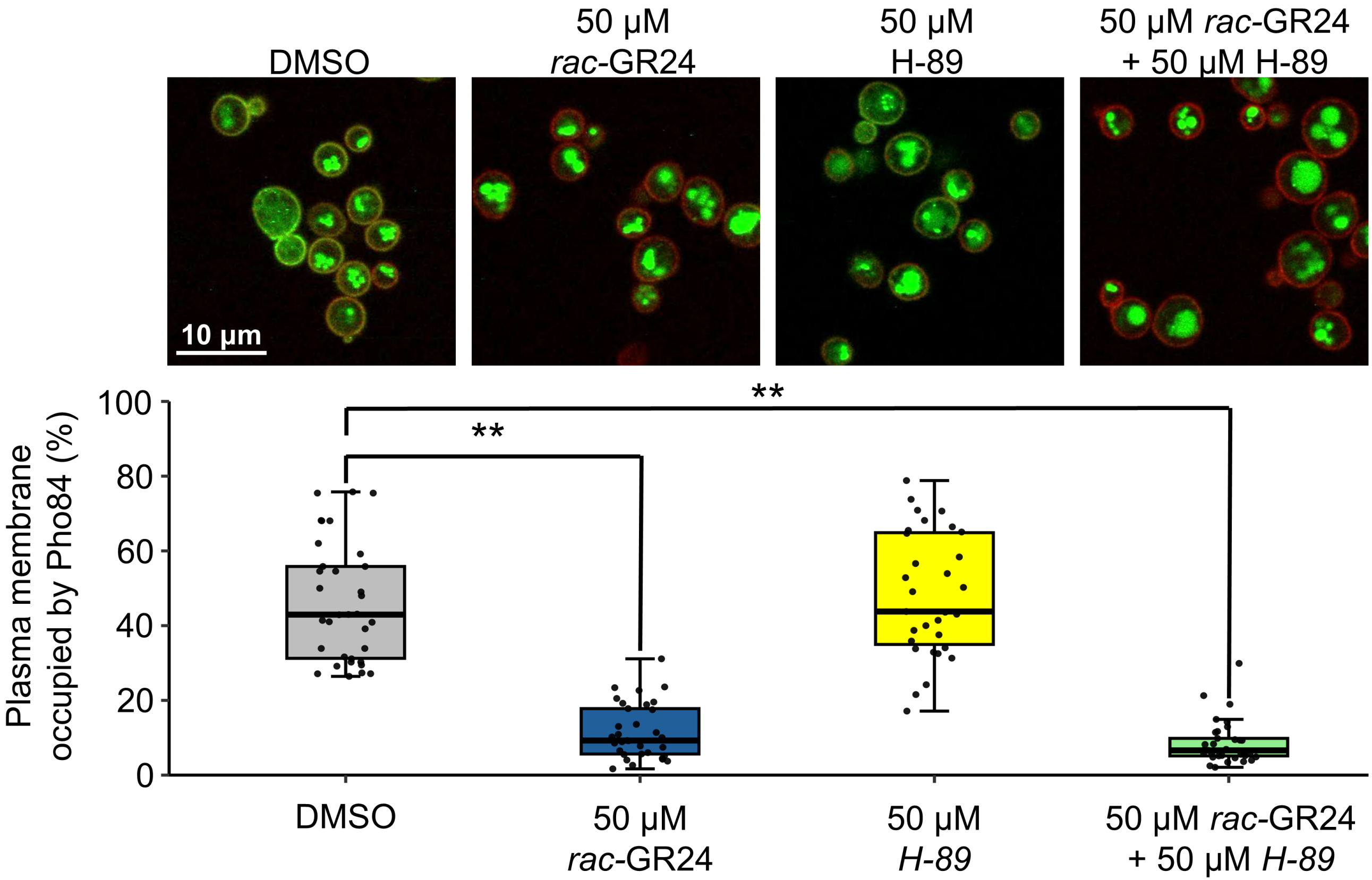

Figure S7. PKA inhibitors do not perturb strigolactone-mediated internalization of Pho84. Pho84-GFP and 2xPH-ymScarlet co-localization upon four hours of Pi starvation followed by DMSO, 50 μM *rac*-GR24, 50 μM H-89, or 50 μM *rac*-GR24 and 50 μM H-89 treatment for two hours. Each individual datapoint represents a single yeast cell. Two-tailed t-test was used to measure statistical significance between DMSO and treatment of ligand ** *p value* < 0.005.

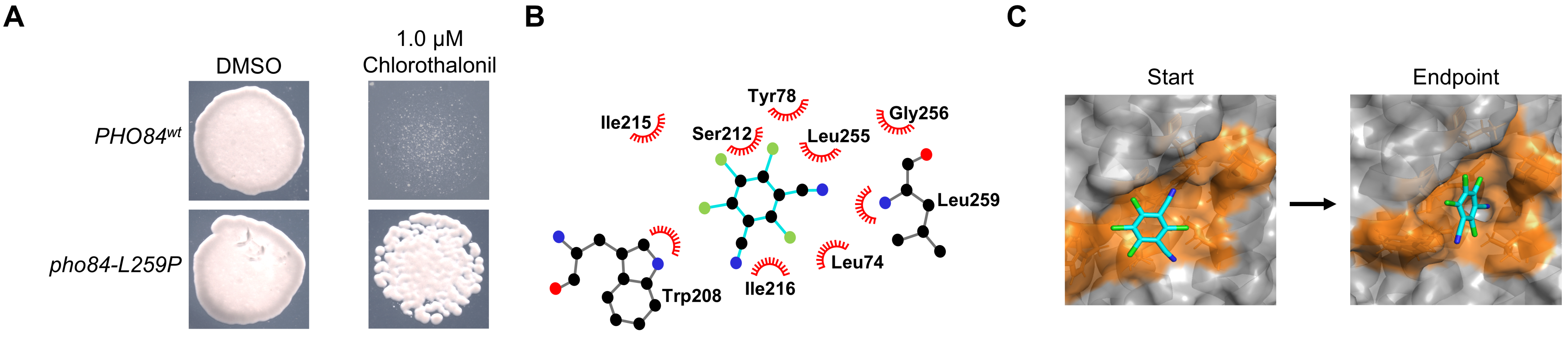

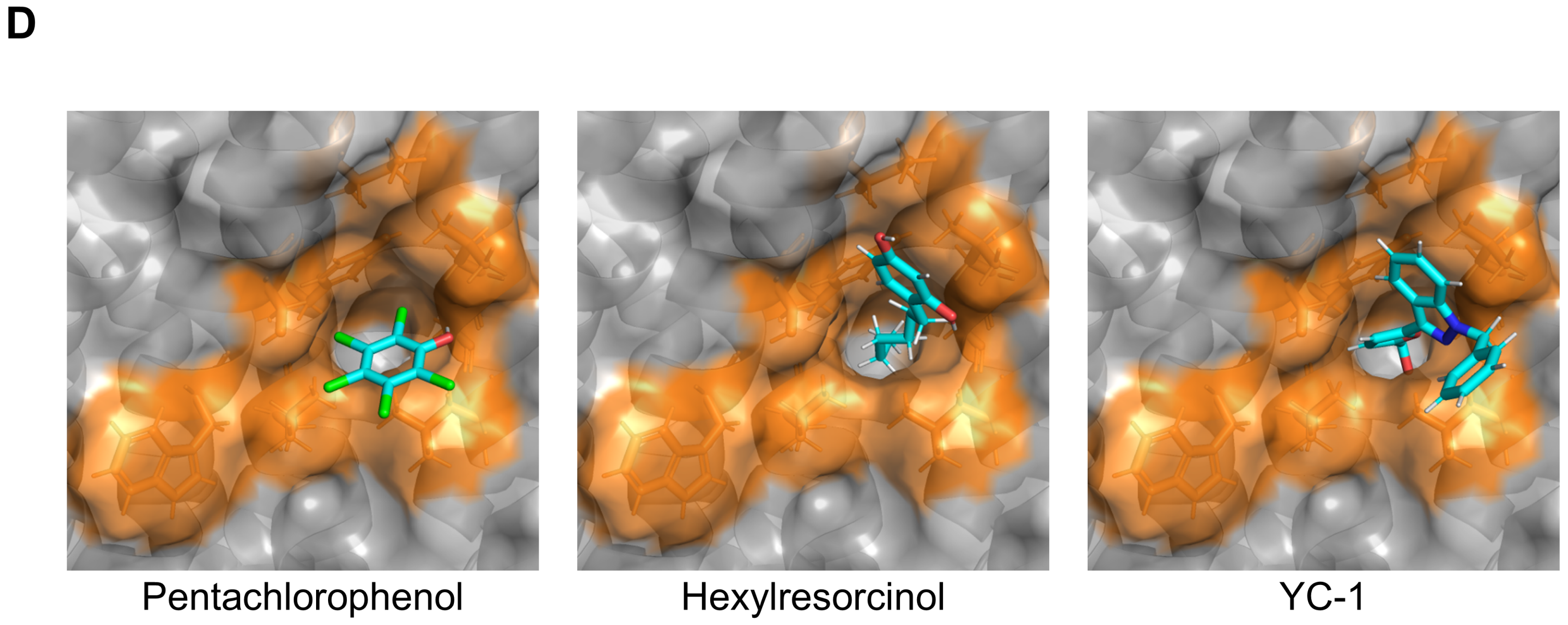

Figure S8. Pho84-perurbagens bind into the transmembrane pocket

(**A**) wild-type PHO84 (top) and *pho84-L259P* (bottom) strains treated with 1.0 µM Chlorothalonil. (**B**) Representation of residues on Pho84 that interact with chlorothalonil after molecular dynamics simulations. (**C**) CTN interacts with the SL binding pocket. Start and endpoints of Pho84 MD simulation with simulation with CTN present. (**D**) Pho84-perturbagens docked into an open pose of the Pho84 transmembrane pocket
