# Supplementary material for: Strigolactones and antifungals induce internalization of major facilitator superfamily transporters
Source: J Biol Chem. 2026 Oct 9;302(12):113631. doi: 10.1016/j.jbc.2026.113631 (PMC13769216; doi:10.1016/j.jbc.2026.113631)
Supplement: Supplementary file 2 [file mmc3.docx]

**Supporting Movie S1. Stabilization of a cryptic pocket in the transmembrane domain of Pho84.** This movie depicts the first 300 ns of two representative MD trajectories for a Pho84 structure embedded in a DMPC bilayer in the presence of no ligand (left) and in the presence of +GR24 (right). Pho84 is shown in a grey transluscent surface depiction with predicted ligand interacting residues coloured in orange. +GR24 is depicted in cyan. For clarity ions, water, and the DMPC bilayer were omitted from this visualization. A graph of ligand-binding pocket volume is depicted on the right.

**Supporting Movie S2. Altered intracellular domain dynamics in response to binding of +GR24 in the transmembrane domain.** This movie depicts 1 μs of two representative MD trajectories for a Pho84 structure embedded in a DMPC bilayer in the presence of no ligand (left) and in the presence of +GR24 (right). Pho84 is shown in a grey translucent surface depiction with predicted ligand interacting residues coloured in orange, the N-terminal intracellular region coloured in magenta, the intracellular loop coloured in green, and the C-terminal intracellular region coloured in yellow. +GR24 is depicted in cyan. For clarity, ions, water, and the DMPC bilayer were omitted from this visualization.
